# A Generic LC-HRMS Screening Method for Marine and Freshwater Phycotoxins in Fish, Shellfish, Water, and Supplements

**DOI:** 10.3390/toxins13110823

**Published:** 2021-11-22

**Authors:** Mirjam D. Klijnstra, Elisabeth J. Faassen, Arjen Gerssen

**Affiliations:** 1 Wageningen Food Safety Research, Wageningen University and Research, Akkermaalsbos 2, 6708 WB Wageningen, The Netherlands; els.faassen@wur.nl (E.J.F.); arjen.gerssen@wur.nl (A.G.); 2 Aquatic Ecology and Water Quality Management, Wageningen University and Research, Droevendaalsesteeg 3a, 6708 PB Wageningen, The Netherlands

**Keywords:** phycotoxins, screening, high resolution mass spectrometry, HILIC, reversed phase, shellfish, water, food supplements

## Abstract

Phycotoxins occur in various marine and freshwater environments, and can accumulate in edible species such as fish, crabs, and shellfish. Human exposure to these toxins can take place, for instance, through consumption of contaminated species or supplements and through the ingestion of contaminated water. Symptoms of phycotoxin intoxication include paralysis, diarrhea, and amnesia. When the cause of an intoxication cannot directly be found, a screening method is required to identify the causative toxin. In this work, such a screening method was developed and validated for marine and freshwater phycotoxins in different matrices: fish, shellfish, water, and food supplements. Two LC methods were developed: one for hydrophilic and one for lipophilic phycotoxins. Sample extracts were measured in full scan mode with an Orbitrap high resolution mass spectrometer. Additionally, a database was created to process the data. The method was successfully validated for most matrices, and in addition, regulated lipophilic phycotoxins, domoic acid, and some paralytic shellfish poisoning toxins could be quantified in shellfish. The method showed limitations for hydrophilic phycotoxins in sea water and for lipophilic phycotoxins in food supplements. The developed method is a screening method; in order to confirm suspected compounds, comparison with a standard or an additional analysis such as NMR is required.

## 1. Introduction

Phycotoxins such as marine toxins and cyanotoxins are produced by algae and cyanobacteria that are naturally occurring in marine, estuarine, and fresh waters. Phycotoxins can accumulate in various edible marine species such as fish, crabs, and shellfish. In shellfish, toxins accumulate mainly in the digestive glands without causing intoxication to the shellfish itself [1].

The consumption of contaminated fish, shellfish, and other aquatic species may lead to human exposure to phycotoxins. The consumption of food supplements, in which toxin-producing algae or cyanobacteria as well as contaminated fish or shellfish are used, is another possible exposure route. In addition, exposure may occur through direct contact/ingestion of toxic phytoplankton, e.g., during swimming. Exposure to phycotoxins can cause severe intoxication symptoms such as skin irritation, paralysis, diarrhea, and even death [2,3]. The intoxication of humans, cattle, domestic animals, and wildlife has been reported [4,5,6,7,8,9,10], and throughout the world, phycotoxins are held responsible for approximately 60,000 instances of human intoxication every year [11].

Marine toxins are usually classified by the syndromes they cause: paralytic shellfish poisoning (PSP) is caused by saxitoxin (STX) and analogues, diarrheic shellfish poisoning (DSP) is caused by okadaic acid (OA) and dinophysistoxins (DTXs), amnesic shellfish poisoning (ASP) is caused by domoic acid (DA), and neurotoxic shellfish poisoning (NSP) is caused by brevetoxins (PbTxs) [12]. Other phycotoxins include azaspiracids (AZAs), yessotoxins (YTXs), cyclic imines (CIs), pectenotoxins (PTXs), microcystins (MCs), nodularins (NODs), cylindrospermopsins (CYNs), anatoxins (ATXs), tetrodotoxins (TTXs), ciguatoxins (CTXs), palytoxins (PlTXs), and β-N-methylamino-L-alanine (BMAA). AZAs cause diarrhea; PTXs, CYNs, MCs, and NODs are hepatotoxic; CIs, ATXs, TTXs, and BMAA are neurotoxic; CTXs show gastrointestinal and neurological symptoms, and PlTXs cause gastrointestinal problems or respiratory distress [13,14,15,16,17,18,19]. Some toxins, such as TTXs and PbTxs, can cause fatal intoxication in humans at relatively low doses [20]. YTXs are lethal to mice after intraperitoneal injection, but not for humans after the consumption of contaminated shellfish [21]. All mentioned toxin groups contain multiple analogues; the largest group are the MCs, with more than 160 different structures reported in the literature and registered in Scifinder [22,23,24]. Some analogues are more toxic than others, and therefore for some phycotoxin groups, toxicity equivalent factors (TEF) have been established.

Less than ten percent of all phycotoxins described in the literature are available as a (certified) standard. Standards are isolated from contaminated shellfish or algae, or are chemically synthesized. There are only a few producers that sell certified phycotoxin standards. Furthermore, the availability of contaminated shellfish and algae is limited, and it is time-consuming to produce such purified standards, which makes standards relatively expensive [25].

From an analytical perspective, phycotoxins can be divided into two groups based on their polarity. The lipophilic phycotoxins include compounds such as OA, DTXs, AZAs, PTXs, and YTXs, while DA, STXs, and CYNs are examples of more hydrophilic toxins. The analysis of lipophilic and hydrophilic compounds requires different approaches. Lipophilic compounds tend to dissolve better in organic solvents and are separated under reversed phase conditions. Hydrophilic compounds dissolve better in water and are preferably separated by Hydrophilic Interaction Liquid Chromatography (HILIC) or are derivatized to reduce polarity and make reversed phase separation possible [26]. Differences in polarity also affect the extraction; lipophilic toxins can generally be better extracted from less polar solvents than hydrophilic toxins. Structures of toxins of which the standards used in this study are given in S1.

The large number of known toxins, combined with the wide range of chemical properties of these toxins and the lack of analytical standards, makes it difficult to develop one single method that can unambiguously identify all toxins. However, in certain cases such as a food intoxication event, a broad screening method to find the cause of the intoxication can be valuable.

In the past, bioassays such as mouse and rat bioassays were common methods for the screening of marine toxins. Animals were fed shellfish or injected intraperitoneally with shellfish extracts, and diarrhea or death were the endpoints [27,28]. Some disadvantages of these tests include the fact that the animal’s response to possible phycotoxins is difficult to extrapolate to humans and that the resistance to the use of experimental animals is growing [29]. The main advantage of animal testing is that the selectivity is low; therefore, the assays may also be sensitive towards possible unknown toxins. Alternative, widely used screening approaches are immunoassays such as enzyme-linked immunosorbent assays and lateral flow immunoassays [30], receptor binding assays [31,32], and cell-based invitro assays [33]. Most of these tests are specific for certain toxin groups, which makes them suitable for finding which toxin group caused a specific intoxication. However, to identify and quantify the causative toxin itself, these approaches are less suitable. At present, instrumental methods such as LC-MS/MS are widely used in toxin analysis due to their high sensitivity and selectivity [34]. However, these methods are also only dedicated to a relatively small group of toxins and/or a specific matrix, which makes them unsuitable as a broad screening method. LC coupled with high resolution mass spectrometry (LC-HRMS) has the advantage of being able to screen for a very large number of compounds per run. LC-HRMS allows for an untargeted analysis with the possibility to detect toxins retrospectively, which is not possible for LC-MS/MS analysis. It has been shown that LC-HRMS is a useful technique for screening food samples to confirm the presence of a high variety of analytes. For phycotoxins, a few methods are described using LC-HRMS, and these are all only suitable for lipophilic or hydrophilic toxins [35,36,37,38,39,40]. Broad screening LC-HRMS methods provide new opportunities for toxin analysis in food and water; however, they also bring new challenges. The number of analytes that can be detected is too large to process and verify manually, which is how it is typically completed for quantitative LC-MS/MS methods. Therefore, databases with target analytes and sufficient information as retention and/or fragmentation information are needed to facilitate automated identification. When such a database is available, the extraction of the analytes of interest from the raw data can be automated. Another challenge is the extraction of the analytes of interest from the sample matrix and the LC separation, as outlined below.

For the hydrophilic compounds, the extreme polar ones are typically separated by hydrophilic interaction liquid chromatography (HILIC). Various types of columns such as zwitterion or amide-based columns [41,42,43] are used for PSP toxins. Alternative approaches for these toxins are ion-pair separation [44,45] and pre-column derivatization [46,47]. Furthermore, TTXs can also be separated by HILIC [48]. For some hydrophilic compounds which are somewhat less polar, such as CYNs, a reversed phase separation can also be successfully applied [7,49,50]. In general, HILIC separation as compared to reversed phase separation is more sensitive to mobile phase and matrix composition, which may cause shifts in retention time.

Lipophilic toxins are separated under reversed phase conditions. DSP toxins can be combined with PTXs, YTXs, AZAs, and CIs or with MCs and NOD in one single method for lipophilic toxins. Acidic, neutral, or alkaline reversed phase chromatographic conditions are used in combination with a C_8_ or C_18_ column for separation [34,51,52]. For MS analysis, OA, DTXs, and YTX are preferably analyzed in the negative ionization mode to obtain better sensitivity, while AZAs and CIs are preferably analyzed in the positive ionization mode.

Existing methods described for MCs are mostly suitable for water samples. MCs can be separated under acidic conditions in combination with a C_18_ column, and are analyzed in the positive ionization mode [8,53]. PlTXs, PbTxs, and CTXs are similar to MCs that have been separated under acidic conditions in combination with a C_18_ column and are also analyzed in the positive ionization mode [54,55,56]. DA is retained on reversed phase columns as well as on HILIC columns; therefore, DA is often included in multi-toxin methods for DSP or PSP toxins and can be analyzed in the positive and the negative ionization mode [57,58].

A final challenge in developing one single method for toxins is that some toxins might present as protein-bound or metabolites in the samples. To include these forms, additional sample preparation steps are usually required. For example, shellfish tend to form a wide variety of esters from PbTxs, CIs, PTXs, OA, and DTXs, from C_16_ to C_24_ chains, and with different saturations [59,60,61,62,63,64]. These esters can be analyzed intact [63], however, it is impossible to have standards for all of them. Therefore, esters are often transformed into their deconjugated forms by alkaline hydrolysis [65]. MCs can bind to different thiol-containing compounds [66,67]; the analysis of bound MCs is laborious and often leads to the loss of structural information [68,69]. Moreover, BMAA can be present in these forms, and releasing BMAA from these forms would require hot acid hydrolysis [70,71], which is not compatible with most other toxins. It will be impossible to overcome all these challenges; however, libraries can be updated and the existing data can be reprocessed using retrospective data analysis.

In this study, we investigated the possibilities of using LC-HRMS as a generic technique to screen for a broad variety of phycotoxins in different matrices such as fish, shellfish, freshwater, seawater, and food supplements. This method could be applied, for instance, in case of an incident where symptoms cannot be directly related to regulated phycotoxins. The aim was to develop a method of extracting different sample types with one generic extraction method, and to analyze sample extracts with one LC method for hydrophilic and another LC method for lipophilic phycotoxins. In order to process the data, a database was created from the literature.

## 2. Results and Discussion

**Figure 1:**
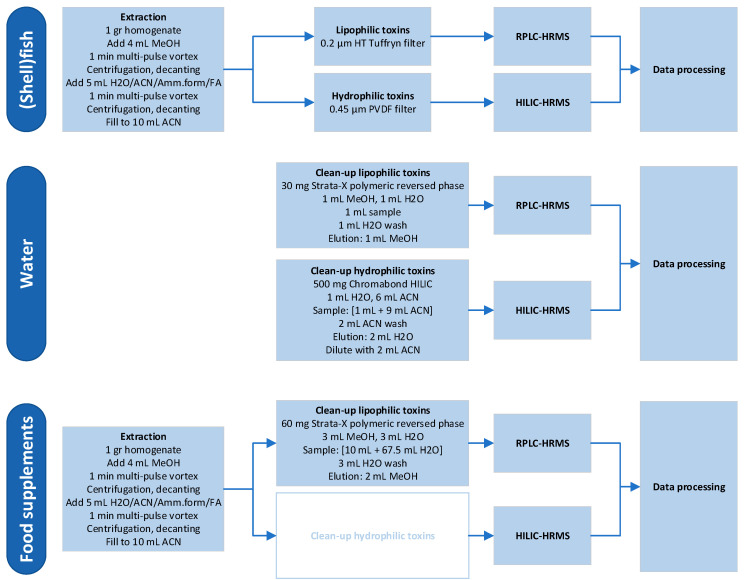
Workflows for the different matrices and toxin groups. All steps were developed and optimized during this research, except for the clean-up of hydrophilic toxins in food supplements (shown in the white text box).

For method development and the optimization of the extraction procedures described in Section 2.1 Section 2.2 and Section 2.3, extracts were analyzed with targeted LC-MS/MS. The optimized extraction procedure was then subsequently used in the LC-HRMS method development. For the different matrices and toxin groups, different workflows were applied. These are displayed in Figure 1. All steps were developed and optimized during this research, except for the clean-up of hydrophilic toxins in food supplements.

### 2.1. Extraction of Shellfish Samples

To develop an extraction method for both lipophilic and hydrophilic phycotoxins in shellfish and fish tissue, several extraction techniques, volumes as well as the duration of the extraction were tested. Natural contaminated shellfish samples were used containing the following lipophilic toxins: OA, dinophysistoxin-2 (DTX2), DTX3 (fatty acid ester of OA 16:0 (number of carbon in the fatty acid chain length: number of double bonds)), azaspiracid-1 (AZA1), AZA2, AZA3, and 13-desmethyl spirolide C (SPX1) as well as samples containing the hydrophilic toxins, PSP toxins.

**Figure 2:**
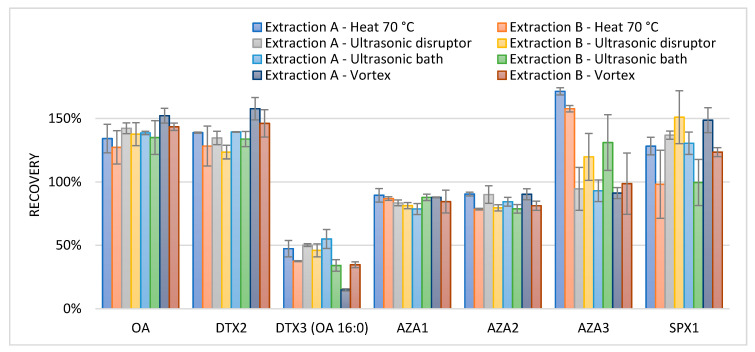
Average relative recoveries (*n* = 2, error bars represent the min and max values) of lipophilic phycotoxins with various extraction methods, compared to a triple extraction with methanol and vortex mixing. Extraction A: 4 mL methanol (MeOH), followed by the second step with 4 mL water/acetonitrile/ammonium formate/formic acid (H_2_O/ACN/Amm.form/FA) (55:45 *v*/*v*, 2 mM, 0.5 mM). Extraction B: 4 mL H_2_O/ACN/Amm.form/FA (55:45 *v*/*v*, 2 mM, 0.5 mM), followed by the second step with 4 mL methanol.

For the extraction, two different options for the extraction solvents were used. Extraction A: the first extraction step was carried out with 4 mL methanol (MeOH), and the second step with 4 mL water/acetonitrile/ammonium formate/formic acid (H_2_O/ACN/Amm.form/FA) (55:45 *v*/*v*, 2 mM, 0.5 mM). Extraction B: the first step was performed with 4 mL H_2_O/ACN/Amm.form/FA (55:45 *v*/*v*, 2 mM, 0.5 mM), followed by the second step with 4 mL methanol. Methanol was meant to extract the more lipophilic phycotoxins, and H_2_O/ACN/Amm.form/FA was meant to extract the more hydrophilic phycotoxins. To test various extraction methods after the addition of each extraction solvent (Extraction A and B), the samples contaminated with lipophilic toxins were treated differently. Samples were either vortex-mixed for 1 min, placed in an ultrasonic bath for 5 min, ultrasonic disrupted for 30 s at 11 W (RMS), 40 kHz, or heated for 5 min at 70 °C, all followed by centrifugation and decanting of the extract. To obtain the recovery data for the lipophilic toxins, the results were compared to those of an interlaboratory validated confirmation method for lipophilic phycotoxins [72], which is a triplicate extraction with 3 mL methanol, followed by vortex mixing for 1 min, and the centrifugation and decanting of the extract, adding methanol after each time. The extract was complemented with methanol up to 10 mL and filtered before analysis. Relative recoveries are shown in Figure 2.

The applied extraction methods showed recoveries from 78% to 156% for the lipophilic phycotoxins when compared to a triple extraction with methanol; except for DTX3, which had an average recovery of 40%, and AZA3, which presented an average of 165% when heated. On average, the recovery seemed improved and the range (min and max recovery) was lower when starting the extraction with methanol (extraction A). The lower recovery for DTX3 can be explained by the presence of fatty acid ester chains in these molecules. To improve the extraction recovery for DTX3, multiple extraction steps with methanol are required [36]. The recovery of AZA3 after heat extraction was higher than 100%. Besides AZA1, 2, and 3, over 60 azaspiracid analogues are described in the literature. It is known that AZA17 can be heat transferred to AZA3 [73], therefore AZA17 was added to the LC-MS method. The observed high recoveries for AZA3 were indeed caused by this transformation. In order to avoid the thermal degradation of the toxins, heating was not chosen as an extraction method. Based on the obtained results for the lipophilic toxins, Extraction A was chosen and a combination of first ultrasonic disruption and second vortex mixing was used as the extraction technique.

In order to keep the extraction procedure as generic as possible, only minor changes in extraction volumes were tested for the hydrophilic phycotoxins. For these experiments, shellfish samples contaminated with PSP were used. Extraction A was used, as described before; for the first step, after the addition of methanol, the sample was ultrasonically disrupted, and for the second step, after the addition of H_2_O/ACN/Amm.form/FA (55:45 *v*/*v*, 2 mM, 0.5 mM), the sample was vortex-mixed. The amount of extraction solvent used in the second step was changed from 4 to 5 mL. After the second extraction, the combined extracts were complemented with acetonitrile up to 10 mL instead of methanol in order to increase solvent compatibility with the HILIC separation. To obtain recovery data, the results of these experiments were compared to the results of a triplicate extraction with 3 mL H_2_O/ACN/Amm.form/FA (55:45 *v*/*v*, 2 mM, 0.5 mM). All extracts were complemented with acetonitrile up to 10 mL and then filtered. Relative recoveries when changing the extraction solvent from 4 to 5 mL did not improve. Compared to the triple extraction, the relative recoveries of the individual toxins were between 55% and 126%. These recoveries are acceptable as the developed method’s purpose is to conduct a broad screening in case of an incident such as an intoxication, and not to be used as enforcement in official control.

**Figure 3:**
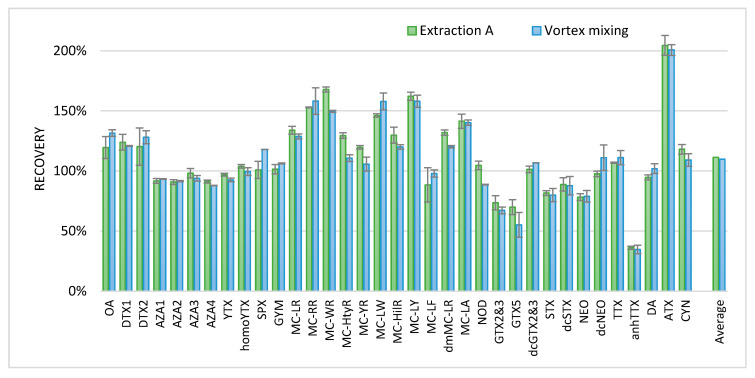
Average relative recoveries (*n* = 2, error bars represent the min and max values) of toxins with two extraction methods. Recoveries of the tested extractions are compared to those of three different in-house methods (see text).

To verify the recovery of the phycotoxins, blank shellfish samples were fortified with the available standards. Furthermore, a similar extraction that was produced solely by vortex mixing instead of ultrasonic disruption was added to make the method more efficient. To obtain the recovery data, the results were compared to the results of the confirmation methods available in the laboratory for the specific phycotoxin classes. The extraction procedures for these confirmation methods are slightly different for the various toxin classes as they are developed and optimized for a specific toxin class. For the lipophilic marine toxins, a triplicate extraction with methanol was applied; for the microcystins (MCs), a single extraction with 6 mL H_2_O/ACN/FA (25:75:1 *v*/*v*) followed by a hexane partitioning step was applied; and for the hydrophilic toxins, a double extraction with H_2_O/MeOH/HAc (50:50 *v*/*v*, 15 mM) was applied [34,41]. Relative recoveries are shown in Figure 3.

All relative recoveries were between 80% and 120%, with some exceptions. For some PSP toxins (GTX5 and GTX2 and 3) and anhydroTTX (anhTTX), recovery seemed lower compared to the in-house confirmatory methods used. This might be explained by the variation of matrix effects. For some toxins, a relatively high recovery was observed, such as for the MCs and ATX. The extraction using the ultrasonic disruptor and vortex mixing (extraction A) and that using solely vortex mixing seemed not to differ. Therefore, to further improve the ease of use of the method, for the analysis of shellfish and fish material, ultrasonic disruption was replaced by vortex mixing. For screening approaches of tissues other than shellfish and fish, this procedure will most probably also be applicable; however, this was not investigated. The final procedure with extraction A and only vortex mixing is described in the materials and methods chapter.

### 2.2. Clean-Up of Water Samples

In water samples, the phycotoxins can be present as dissolved toxins. However, if the samples contain toxic algae, as is often the case in surface water samples, most of the toxins are expected to be present within the algal cells. As both the dissolved and the intracellular toxins need to be analyzed, the algal cells were disrupted to release the intracellular toxins. After disruption, all toxins are expected to be present in the supernatant, which can then be processed further. Different disrupting methods were tested with a laboratorial culture of *Alexandrium Ostenfeldii* from the Ouwerkerkse Kreek in The Netherlands, which is a water sample containing algal cells that produce SPX1 and gymnodimine (GYM) [74].

Samples were ultrasonically disrupted, placed in an ultrasonic bath, frozen, ground, or used without any treatment. After the treatment, the intact cells and their corresponding intracellular toxins were removed using a 0.2 µm filter. Through this, the efficiency of the disruption method could be determined. Furthermore, water samples with algal cells were filtered without cell disruption. These filters with intact cells were washed with 5 mL water to determine any osmotic effects. Additionally, filters with intact cells were washed with 1 mL methanol to determine if methanol could release the intracellular toxins.

**Figure 4:**
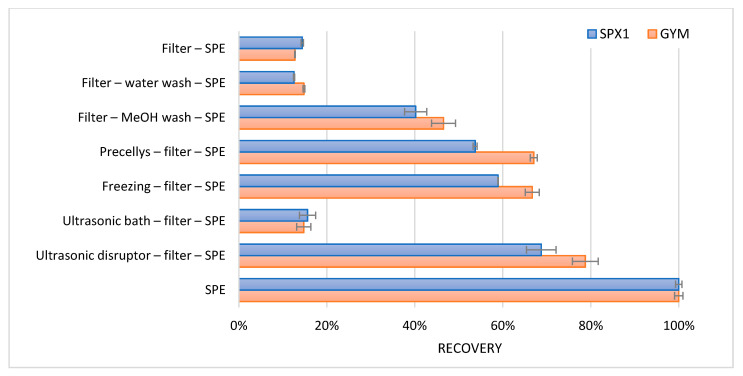
Average relative recoveries (*n* = 2, error bars represent the min and max values) of SPX1 and GYM after algal disruption. Recovery of the SPE treatment is set at 100%.

To concentrate the toxins after the various treatments, a generic reversed phase solid phase extraction (SPE) was carried out. A total of 1 mL of the filtered water samples after disruption was applied onto the SPE. To reduce the organic strength and provide for the retention of SPX1 and GYM on the SPE cartridge, the methanol filter wash was diluted with 4 mL water. Likewise, a sample without any treatment or filtration step was applied onto the SPE. Precellys is a homogenizer employed through bead beating. The SPE procedure used is as follows: a 30 mg polymeric reversed phase cartridge was activated and conditioned with 1 mL methanol, followed by 1 mL water. A total of 1 mL of the sample was loaded onto the cartridge, and the cartridge was washed with 1 mL water. Subsequently, SPX1 and GYM were eluted with 1 mL methanol. Because the concentration of SPX1 and GYM in the water sample was unknown, the result with the highest response was set as 100% recovery. All other results were relative to this. The observed apparent recoveries are shown in Figure 4.

Only a small amount (14%) of the total SPX1 and GYM was present in an extracellular form. Washing with methanol, grinding with the Precellys, freezing, and ultrasonic disruption caused some release of the intracellular toxins. However, the best results were achieved with the use of only the SPE clean-up. The largest amount of toxins were released when the water with the algal cells were added directly onto the SPE cartridge. The cells were most probably lysed during the addition of methanol.

**Figure 5:**
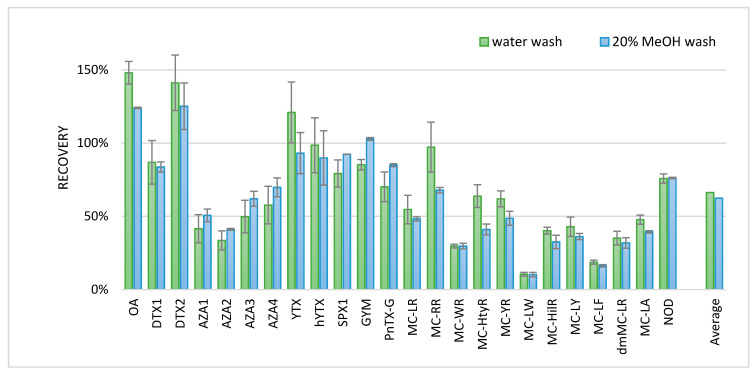
Average recoveries (*n* = 2, error bars represent the min and max values) of lipophilic phycotoxins in water after solid phase extraction.

Brackish medium was used to cultivate the strain of *Alexandrium Ostenfeldii*. Therefore, to test the SPE procedure for other lipophilic phycotoxins, a blank brackish medium was fortified with various lipophilic phycotoxins to imitate brackish water conditions. Two different washing steps were tested to improve the method. After loading the water sample onto the cartridge, a wash with either 1 mL H_2_O or H_2_O/MeOH (80:20 *v*/*v*) was applied, and the toxins were subsequently eluted with 1 mL methanol. The more methanolic wash was used to potentially remove more interferences. The results of the SPE experiments were compared to the results obtained with a standard solution. Results are shown in Figure 5, where the measurement of the standard solution is set at 100% recovery.

Besides the toxins in Figure 5, DA was also analyzed but not recovered. DA was probably not retained on the SPE cartridge, since DA was also not well-retained on the reversed phase LC column with the conditions of this developed screening method. Recoveries of some of the more hydrophobic MCs were low (10–19%). Some of the other toxins gave a recovery > 100%; all of these compounds were measured in the negative ionization mode. Although matrix effects are generally less pronounced in the negative ionization mode, the enhancement of these compounds could be due to the matrix effects. The MCs tested seemed to have a slightly better recovery when washed with water as compared to 20% MeOH. Therefore, a wash with water was included in the final procedure for lipophilic phycotoxins in the water samples. The final procedure, without cell lysing with a water wash before the SPE procedure, is described in the materials and methods chapter.

**Figure 6:**
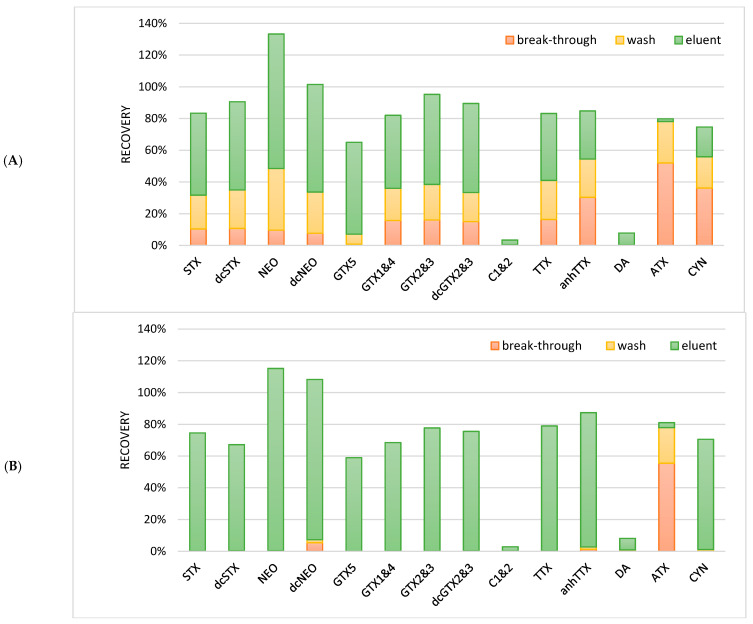
Average recoveries (*n* = 2) of hydrophilic phycotoxins by HILIC solid phase extraction with (**A**) a sample load with 75% organic strength and (**B**) a sample load with 90% organic strength. The recovery is absolute and total recovery should be 100%.

For the hydrophilic phycotoxins, a different approach was needed because hydrophilic phycotoxins are not retained on the SPE cartridge under reversed phase conditions and are therefore eluted simultaneously with the salts present in the sample. These salts would interfere with the applied LC method. This LC method is based on HILIC. HILIC starts with a high ratio of organic solvent, and water content increases during the gradient. Under HILIC conditions, the toxins of interest should also be present at a high organic concentration in order to retain them in the column. Therefore, to increase sensitivity and to decrease solvent polarity the sample was evaporated and reconstituted in a smaller volume of ACN. In order to investigate this possibility, 100 mL of brackish water sample containing PSP toxins [74] was evaporated to dryness with the use of a rotavapor, and then reconstituted in 10 mL solvents of different organic strengths: ACN:H_2_O (90–75% *v*/*v*) containing amm.form/FA (2 mM, 0.5 mM). Due to the high salt content in the brackish water sample, two immiscible layers were formed; these samples could not be used for the LC-MS/MS analysis. Different dilution volumes for reconstitution were tested, but without success; up to 60 mL of the immiscible layers could still be observed. As an alternative, the SPE procedure with an HILIC cartridge was investigated. A total of 1 mL of the blank brackish water samples was fortified with 100 ng/mL of the hydrophilic phycotoxins: PSP toxins, TTXs, DA, ATX, and CYN. The fortified sample was either diluted with 3 mL acetonitrile (75%) or 9 mL acetonitrile (90%) in order to test the organic strength needed for the toxins to be retained on the SPE cartridge. After the activation and equilibration of a Chromabond HILIC cartridge, the diluted water sample was loaded onto the cartridge and subsequently washed with 2 mL ACN. The hydrophilic phycotoxins were eluted with 2 mL water. The breakthrough of the applied sample extract and the washing solvent were also collected. In order to be able to analyze these extracts, the organic strength of the solution should be large. Therefore, the breakthrough (water) of the sample was evaporated and reconstituted in 75% acetonitrile, the washing solvent was diluted with water, and the eluent was diluted with acetonitrile. All fractions were compared to a standard solution to obtain an absolute recovery. The results are given in Figure 6. 

When the sample was diluted to 75% acetonitrile, most hydrophilic phycotoxins were not well-retained, which led to losses during loading and washing (Figure 5). However, when the samples were diluted to 90% acetonitrile, most hydrophilic phycotoxins were well-retained during loading and washing, except for ATX, which was still lost during the loading and the washing step. Furthermore, C1 and 2 as well as DA showed bad recoveries (<8%). Although there is no clear explanation, it might be that these phycotoxins precipitated during the dilution of the sample together with the salts, were not eluted from the cartridge (due to charge/pH during sample loading), or were suppressed due to the matrix effects during the LC-MS/MS analysis. With broad screening methods, it will be difficult to have optimized conditions for each individual toxin or toxin group. Especially under HILIC conditions, the optimization will be difficult as many parameters such as the type of HILIC, salts, pH, and organic strength strongly influence the retention mechanism. Therefore, the clean-up procedures in this approach is a compromise between covering as many toxins as possible, a sufficient sample clean-up, and a concentration of the sample. The final procedure with a sample load of 90% organic strength is described in the materials and methods chapter.

### 2.3. Extraction and Clean-Up of Food Supplements

There is a large variety of food supplements that can potentially contain phycotoxins. These supplements can, for example, be based on algae, algal oils, fish, fish oils, or freeze-dried fish or shellfish. The supplements can be in the form of oils, pills, or powders. It can be expected that the extraction of toxins for this wide variety of matrices would be difficult. It will be impossible to obtain good recoveries for all available types of supplements. With the experience of developing an extraction procedure for shellfish (Section 2.1) and the water samples (Section 2.2), a procedure for food supplements was developed and tested. A recovery of both the extraction and the SPE was determined by fortifying food supplements before extraction and fortifying another extract of the same product after SPE. Results are shown in Table 1.

As expected, the results differed largely between matrices and toxins. The lowest recoveries were obtained for YTX and hYTX. On average, the recovery was 61%; however, the recoveries ranged from 9% to 102%. Because the tested matrices were very different, it was difficult to develop a generic method that gave acceptable recoveries for all compounds in all types of supplements. With generic clean-up methods such as the SPE clean-up applied for water, covering a wider variety of toxins as well as various types of matrices is a challenge. The SPE procedure can be further optimized to improve recoveries and remove matrix interferences. However, there is the drawback of losing certain toxin classes if the method becomes too specific. Therefore, if food supplements need to be screened for the presence of toxins, additional steps might be incorporated based on the type of matrix in order to assure the applicability of the procedure. For example, standard addition can be applied to determine recoveries for some of the toxins in each individual sample. The approach should be determined per sample and according to the demand of the analysis. A similar approach can be used for the hydrophilic toxins. However, as the HILIC clean-up would be much more sensitive to the variety of matrices used as food supplements, it was decided not to test the approach for the hydrophilic toxins.

### 2.4. Chromatography

#### 2.4.1. Reversed Phase Liquid Chromatography

**Figure 7:**
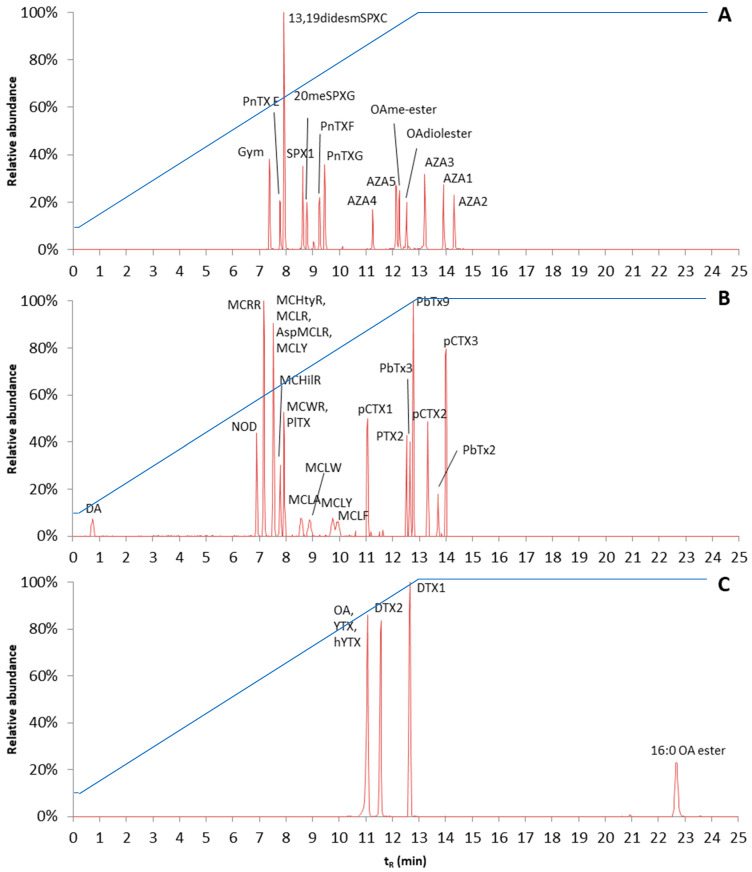
Reconstructed chromatograms of the applied reversed phase liquid chromatography of (**A**) azaspiracids, spirolides, pinnatoxins, gymnodimine, and okadaic acid esters measured in the positive ionization mode; (**B**) domoic acid, microcystins, ciguatoxins, brevetoxins, pectenotoxin, and palytoxin measured in the positive ionization mode; (**C**) yessotoxins, okadaic acid, and dinophysistoxins measured in the negative ionization mode. The blue line is % of mobile phase B.

For the separation of lipophilic phycotoxins, an ACQUITY BEH C_18_ 1.7 µm, 100 · 2.1 mm column was used. Mobile phases and other LC settings were similar to a screening method for pesticides [75], except for mobile phase B. To elute all compounds of interest, and especially the DTX3 toxins (fatty acid esters of OA, DTX1, and DTX2), methanol/water/amm.form/FA (95:5 *v*/*v*, 2 mM, 0.5 mM) was replaced by a stronger organic solvent: acetonitrile/water/amm.form/FA (90:10 *v*/*v*, 2 mM, 0.5 mM). In order to test the separation, a mixture of all available phycotoxins was made. However, as the developed method will also be applied to toxins for which no standards are available, a generic LC procedure was needed. Therefore, a slow gradient of organic strength was used. The gradient was linearly increased from 10% mobile phase B to 100% mobile phase B in 12.8 min. A good separation of the various toxins was obtained; however, the fatty acid ester of OA (16:0 OA ester) was still retained in the column. In order to elute this ester, the gradient was extended and 100% mobile phase B was kept for 12 min. As most of the toxins tested started to elute after 7 min, it may be considered if it is possible to start at 10% mobile phase B and increase slowly to 100% mobile phase B. However, as the method was developed to separate and detect toxins for which no standards were available, it was decided to keep the slow gradient in order to retain these or potential unknown toxins. Reconstructed chromatograms of the various toxin mixtures are shown in Figure 7. The reconstructed chromatograms give an overview of the elution order of the different groups of analytes. The final gradient is shown in Figure 7, and other LC settings are described in the materials and methods chapter.

#### 2.4.2. Hydrophilic Interaction Liquid Chromatography

**Figure 8:**
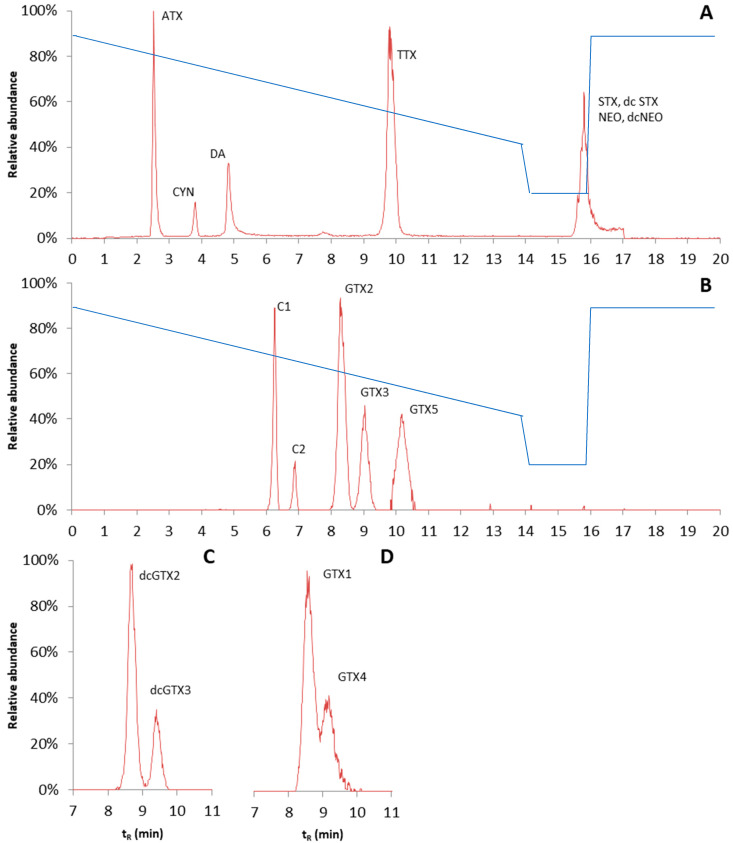
Reconstructed chromatograms of the hydrophilic interaction liquid chromatography of (**A**) anatoxin (ATX), cylindrospermopsin (CYN), domoic acid (DA), tetrodotoxin (TTX), saxitoxin (STX) decarbamoylsaxitixin (dcSTX), neosaxitoxin (NEO), and decarbamoylsaxitoxin (dcNEO) measured in the positive ionization mode; (**B**) N-sulfocarbamoylgonyautoxin 2 (C1) and 3 (C2), gonyautoxin 2, 3, and 5 (GTX2, 3, and 5), measured in the negative ionization mode; (**C**) decarbamoylgonyautoxin 2 and 3 (dcGTX2 and 3) measured in the negative ionization mode; (**D**) gonyautoxin 1 and 4 (GTX1 and 4) measured in the negative ionization mode. The blue line is % of mobile phase B.

For HILIC methods in general, it is known that the matrix has a major influence on sensitivity and retention, especially when samples are treated with a non-selective extraction method [76]. To obtain the sufficient separation of hydrophilic phycotoxins, two different HILIC columns were tested: the Nucleoshell HILIC 3 µm, 100 · 2.7 mm HPLC column (Macherey-Nagel, Düren, Germany) and the TOSOH Bioscience TSKgel Amide-80 2 µm, 150 · 3 mm HPLC column (Tosoh Bioscience, Tokyo, Japan). The properties of the columns are quite different; the Nucleoshell HILIC column is an ammonium sulfonic acid zwitterionic column, while the TOSOH TSKgel amide column consists of silica particles bound with carbamoyl groups. The mobile phase composition was kept similar to that under the reserved phase conditions. However, this time, a slow gradient was initiated with high organic strength (mobile phase B), and a long equilibrium time of 3.9 min was applied. Under the conditions tested, the TSKgel amide column gave much better retention than the Nucleoshell HILIC. However, with the amide column, the obtained chromatographic peaks were still relatively broad, and for some of the toxin isomers, i.e., GTX1 and GTX4, no baseline separation was obtained. Peak shapes of diaminobutyric acid (DAB) and BMAA were poor and therefore sensitivity was low. Hence, DAB and BMAA were excluded from the validation. The chromatograms are shown in Figure 8. The reconstructed chromatograms give an overview of the elution order of the different groups of analytes. The gradient is shown in Figure 8, and other LC settings are described in the materials and methods chapter.

### 2.5. HRMS Method

**Figure 9:**
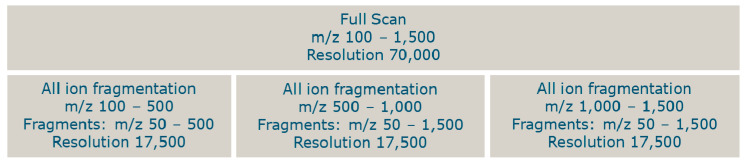
Schematic overview of the HRMS method.

An HRMS method was developed to analyze all toxins from the constructed database (S2). The database contained toxins with molecular masses between 118 and 3380 Da. Assuming that the larger molecules were double or triple charged, all toxins could be measured with a full scan, from *m*/*z* 100–1500 in the positive or negative ionization mode. A larger mass range was not possible due to the calibration mass range of the HRMS. To gain selectivity, fragmentation data was also obtained. Due to the cycle time, it was not possible to insert too many scan events for fragmentation. Each scan event reduces the number of data points per chromatographic peak. The full scan at a resolution of 70,000 takes 0.5 s and each fragmentation scan event (at a resolution of 17,500) takes 0.125 s. Three scan events for all ion fragmentation were created with the use of a data-independent analysis (DIA), resulting in a total cycle time of approximately 0.875 s. To measure the fragments of precursor ions with *m*/*z* 100–500, the DIA inclusion mass was set at *m*/*z* 300 with an isolation width of 400. In the second scan event, fragments of precursor ions with *m*/*z* 500–1000 were measured with the inclusion mass set at *m*/*z* 750 and an isolation width of 500. In the third scan event, the inclusion mass was set at *m*/*z* 1250 with an isolation width of 500 in order to measure the fragments of precursor ions with *m*/*z* 1000–1500. In all scan windows, the obtained masses were measured from *m*/*z* 50 until 1500 (Figure 9). As each window covers multiple toxins, it will not be possible to set a fragmentation energy (normalized collision energy (NCE)) that is optimal for each individual toxin. Therefore, an NCE was chosen where for the majority of the toxins whose standard is available, some fragmentation data could be observed. In the positive ionization mode, an NCE of 40 was selected for each scan event. In the negative ionization mode, at this NCE, there was a lack of sensitivity for the fragments from the precursor window of *m*/*z* 100–500. Therefore, the NCE was further optimized in negative ionization using the PSP toxins. An optimum NCE of 30 was observed and applied to the scan window *m*/*z* 100–500.

### 2.6. Validation

A validation study was performed as described in the materials and methods chapter. For each extraction or clean-up method, 20 blank samples were fortified with standards. In 95% percent of the samples, the precursor ion and at least one fragment ion of the phycotoxins should be discernible. Moreover, some non-fortified samples were included to determine false positives: the presence of signals at *m*/*z* traces representative of the precursor ions and one of its fragment ions. The data described in 2.6.1–2.6.3 were processed with a database containing only the available standards. The data described in 2.6.4 were processed with the complete database (S2). To be able to quantify and get a good indication of the exposure in an incident, the method was also quantitatively validated. All the results and requirements of the quantitative validation are shown in S3: validation data.

#### 2.6.1. Screening Fish and Shellfish

Overall, the validation results of the screening method for shellfish and fish samples were satisfactory. A total of 42 out of the 49 tested phycotoxins were successfully validated for all samples (Tables S3.1 and S3.2). The exceptions were YTX, OA 16:0 ester, AZA5, C1, C2, CYN, and TTX. For YTX, in 3 out of the 20 samples, both precursor and fragment ions could not be found. For the 16:0 OA ester, due to the relative low sensitivity, only the precursor could be obtained, but not a fragment ion. Fragment ions could not be obtained for AZA5 in two fish samples. For the hydrophilic toxins, C2 was present in the samples at a relative low concentration due to the fact that this toxin was only commercially available in an isomeric mixture with the C1 toxin, where the C1 was present at a much higher concentration than the C2 toxin. Therefore, in six of the samples, the C2 fragments could not be observed. Fragments could not be obtained for CYN in three samples, for TTX in two ensis samples, and for C1 in two mussel samples. Six blank samples were analyzed and used to determine if false positives would be observed. For 12 out of the 49 toxins, peaks were observed in the blank samples that corresponded with the precursor ions of the toxins. One cockle sample was naturally contaminated with some cyclic imines, and fragment ions were also found for SPX1, 20 methyl spirolide G, and pinnatoxin G.

#### 2.6.2. Screening Water

When excluding the hydrophilic toxins in seawater, 43 out of the 49 tested phycotoxins were successfully validated (Tables S3.3 and S3.4). As the number of available brackish water samples was low, artificial brackish water samples were created by mixing the sea and fresh water samples (50:50 *v*/*v*). For all lipophilic phycotoxins, the precursor and fragment ions were found, except for OA C_8_-diol ester, where in four samples no fragment ion was found. For YTX, hYTX, and 16:0 OA ester, no parent ion was found in the majority of the samples and there was no fragment ion in the samples with a parent ion. Most hydrophilic phycotoxins were not found in any of the sea water samples. This was most likely due to the high salt content of these samples. Prior to the SPE, the sea water samples were diluted with ACN. Due to the high salt content, a precipitate was formed during the dilution step with ACN, presumably containing some of the hydrophilic phycotoxins. This can be resolved by diluting the sea water samples first with water (50:50 *v*/*v*), followed by dilution with acetonitrile, which was already tested during the validation due to the lack of brackish water. Furthermore, a parent ion and/or fragment ion for DA was lacking in the majority of the brackish, fresh, and tap water samples due to the lack of recovery on the SPE cartridge. For one sample in C2, no parent ion was found, and for eight other samples, there was no fragment ion due to the lower concentration in the standard mixture. Initial experiments with a 1:1 dilution with water showed that when the protocol was adapted for sea water samples, the method was successful for all hydrophilic phycotoxins, except for DA and C2. Five blank samples were analyzed and used to determine if there would be false positives. One brackish water sample was not blank and contained SPX1 and GYM.

#### 2.6.3. Screening Food Supplements

The validation of the screening method for lipophilic phycotoxins in solid food supplements was unsuccessful. Sample descriptions are given in Table S3.5. In solid food supplements, only 5 out of 33 phycotoxins were successfully validated (Table S3.6). For 13,19didesmSPX1, SPX1, 20MeSPXG, PnTX E, and G, the precursor ion and at least one fragment ion were found in 19 or 20 samples. These toxins are all cyclic imines (CIs), which are the most sensitive compounds in the MS detector due to their amino-containing functional groups. Because of the poor results, the spike level of the MCs was increased from 30 µg kg^−1^ for solid food supplements to 50 µg kg^−1^ for the validation of the screening method for lipophilic phycotoxins in liquid food supplements (sample descriptions in Table S3.5). For liquid food supplements, 9 out of 33 phycotoxins were successfully validated (Table S3.7). For 13,19didesmSPX1, SPX1, 20MeSPXG, GYM, PnTX E, F, and G, MC-RR, and NOD, the precursor ion and at least one fragment ion were found in 19 or 20 samples. In some samples, multiple phycotoxin groups had a low recovery. It seems that method performance depended on the sample matrix. It is recommended to fortify each sample during the analysis in order to determine the recovery per sample.

#### 2.6.4. Target Screening with Database

Blanks included in the validation were also screened against the complete database (S2). When tissue samples were measured with the method for lipophilic phycotoxins, all blank samples as well as the solvents used during clean-up contained a chromatographic peak, with an *m*/*z* equal to the mass of PnTX E amine (*m*/*z* 786.51508). As no standard is available for this toxin, it cannot be confirmed and it is highly unlikely that the interference is PnTX E amine. All samples with a matrix contained multiple peaks, with masses equal to the esterification products of GYM. Blank tissue samples measured with the method for hydrophilic toxins contained mainly compounds with masses equal to ATX- and TTX derivatives, although the retention times did not correspond with the expected retention times of the compounds, and no fragment ions were found.

When the water samples were analyzed with the method for lipophilic phycotoxins, all blanks, including blank chemicals used during clean-up, contained a peak with a mass equal to that of PnTX E amine, similar to the tissue samples. No phycotoxins were found in the blank water extracts measured with the method for hydrophilic phycotoxins.

Once again, for food supplements, all blanks, including blank solvents used during clean-up, contained a peak with a mass equal to that of PnTX E amine. All blank liquid samples and some blank solid samples contained multiple peaks with *m*/*z* equal to the esterification products of GYM, OA, or DTXs.

Besides the masses equal to masses of ATX- and TTX derivatives in tissue samples and esterification products in tissue and food supplements, up to a maximum of twenty other peaks were found based on exact mass and isotopic pattern. However, the majority of these peaks could be ruled out based on an unlikely retention time or poor peak shape. If it was a true sample, only a small number of suspected peaks should have been investigated further. Fragments should be checked when known, and when common toxin-specific fragments are present, this should be confirmed against an analytical standard or NMR, depending on the degree of confidence needed. For the validation, the number of false positives was acceptable.

#### 2.6.5. Quantitation Regulated Toxins in Shellfish

The matrix-matched standards were injected before and after the analysis of the sample extracts. From the matrix-matched standards, calibration curves were constructed. Validation results are shown in Tables S3.9–S3.13. The correlation coefficient of lipophilic toxins and DA complied with the requirements for linearity (>0.99). Not all PSP toxins had calibration curves with a correlation higher than 0.99. STX, NEO, and dcNEO are late-eluting compounds with poor peak shapes. Due to the poor peak shapes, a maximum smoothing level was required to integrate the peaks properly; for this reason, the intensities of those peaks became unreliable. Furthermore, the linearity of GTX1 and 4 and C2 was below 0.99. This was due to the poor separation of GTX1 and 4, in combination with the low concentration of GTX4 and the low concentration of C2. The non-complying correlation of dcGTX2 cannot be explained.

For some compounds, one level of the calibration curve deviated more than 20% from their theoretical concentration. In general, this was the lowest fortified level; for lipophilic toxins, this was the case for DTX2 and YTX, and for hYTX at 250 µg kg^−1^ and for the hydrophilic toxins, this was the case for DA, NEO, dcNEO, GTX1 and 4, dcGTX2, and C2. The maximum drift in sensitivity obtained in an analysis series was 25.3% for NEO, which was below the criteria of 30%.

Interfering peaks in the blank samples should be smaller than 30% of the peaks at LOQ. The LOQ is the lowest fortified level during the validation. In some blanks, the molecular ion of a phycotoxin was found. However, all blanks measured during the validation did not show a fragment ion and were therefore not confirmed. Except for one cockle sample which appeared not to be a blank, just as during the screening, low levels of SPX1, 20 methyl spirolide G, and pinnatoxin G were found.

Accuracy was assessed by the recovery estimation with the extracts that were also used to calculate the repeatability (RSDr). For each analyte, the recovery should be between 70% and 120%. The recovery of lipophilic toxins and DA were satisfactory in general. For the PSP toxins, STX, dcSTX, NEO, dcNEO, GTX1 and 4, dcGTX2, and C2 did have a recovery below 70%. These are the same compounds that did not meet the requirements for the calibration curve.

The repeatability (RSDr) was assessed in 5-fold at two different concentrations: 0.5 and 1 times the target value. In each matrix, the RSDr should be less than or equal to 20% for all compounds, and only one outlier (Grubb’s test) was allowed. C2, YTX, and hYTX at 0.5 times the target value had one outlier, and OA at the target value had one outlier. For lipophilic toxins and DA, all RSDr were below 20%. The RSDr did not comply for STX, dcSTX, NEO, and dcNEO at both spiking levels, dcGTX2 and C1 at 0.5 times the target value and C2 at the target value.

All EU-regulated lipophilic phycotoxins, DA, and some PSP toxins can be quantified in shellfish at 0.5 or 1 times the target value. However, late-eluting PSP toxins had poor peak shapes, which gave difficulties during the processing of the results.

### 2.7. Applicability of the Method

The method is suitable for screening for phycotoxins in case of an incident in which the causative toxin in not directly clear. Contrary to dedicated, targeted MS methods, phycotoxins whose standards are not available can be found when the entire database is used during screening. When a phycotoxin is found with the screening method, it is still considered a tentative confirmation. The fragment ions observed with the DIA approach originate from a mass range of precursor ions. Furthermore, for all toxins whose standards are not available, the retention times are unknown. To confirm the presence of a compound, a standard is needed, or an NMR analysis needs to be performed. In order to indicate a level of confidence to the obtained result for the high resolution mass spectrometry analysis, Schymanski et al. proposed various levels of confidence [77]. For the available standards, the highest level of confidence can be obtained (level 1, confirmed structure by reference standard), and for the compounds in the library for which no standards are available, a maximum of level 2/3 toxins can be identified (probable structure or tentative candidates).

The screening method was validated for most of the toxins in the (shell)fish and water samples. The food supplements did not produce satisfactory results. To discern the phycotoxins in 95% of the food supplements, higher spiking levels were needed, which was impracticable due to availability and the cost of the standards. However, food supplements are not normally taken in large amounts, and high levels of phycotoxins should be present in order for them to be harmful. Taking this into consideration, the method is probably sensitive enough to measure toxic levels of phycotoxins in food supplements. In all matrices, an acceptably low number of false positives were found when a target screening that made use of the database was performed.

The quantitative validation results did not meet all the criteria. As the method will mainly be used in case of incidents where high concentrations are expected, and an indicative value is mostly sufficient, this is a minor issue. At least the method will provide some indications of the levels of known toxins present in the sample. For the lipophilic toxins, this information is more reliable than it is for the hydrophilic toxins.

Compared to a straightforward LC-MS/MS analysis, the sensitivity in this procedure is somewhat lower. The data processing time (and therefore cost) are significantly higher. This is due to the data interpretation, which is much more complicated. However, the method will not be applied on a routine basis; it will only be applied in case of an incident and most likely on a limited number of samples.

## 3. Conclusions

A method was developed for the screening of a wide variety of phycotoxins in fish, shellfish, water, and food supplement samples. One extraction method was used for all toxins. Because of the different chemical properties of lipophilic and hydrophilic phycotoxins, a separate clean-up had to be developed for water and food supplements. Additionally, chromatography had to be developed separately for lipophilic and hydrophilic toxins. A validation study was performed for all matrices as a screening method and/or for the quantitation of EU-regulated phycotoxins in shellfish. The validation of the screening of tissue and water samples was successful, except for hydrophilic phycotoxins in sea water. During validation, it appeared that the method for sea water had to be adjusted slightly due to problems with the high salt content. The recoveries of some lipophilic phycotoxins spiked in food supplements ranged from 9% to 102% due to matrix effects. Therefore, it was not possible to validate the method for lipophilic phycotoxins in solid and liquid food supplements. Based on these outcomes and the knowledge that hydrophilic toxins in general are more difficult, the screening of hydrophilic toxins in food supplements was not validated. For quantitative purposes, the EU-regulated phycotoxins were validated. The lipophilic phycotoxins, DA, and some PSP toxins can be quantified in shellfish at 0.5 or 1 times the target value. However, late-eluting PSP toxins had poor peak shapes and therefore could not be quantified.

The developed screening approach can be used in case of an incident. Depending on the toxin found and if a toxin standard or fragmentation information is known via the library, a higher confidence level can be awarded. For full confirmation, either a reference standard or the isolation of the compound is needed, followed by an NMR.

## 4. Materials and Methods

### 4.1. Chemicals and Standards

Formic acid (FA) (98–100%) and acetic acid (HAc) (100%) were purchased from Merck, Darmstadt, Germany. Ammonium formate (Amm.form) (>97%) was purchased from Sigma-Aldrich, Zwijndrecht, The Netherlands. Acetonitrile (ACN) (Ultra LC-MS), methanol (MeOH) (Ultra LC-MS), and water (Ultra LC-MS) were purchased from Actu-All, Oss, The Netherlands. Ammonium hydroxide (25%) was purchased from VWR international, Amsterdam, The Netherlands.

Tables S4.1 and S4.2 lists all the standards used, abbreviations, concentration or purity, and suppliers, divided into hydrophilic and lipophilic phycotoxins.

For method development, 5 mixtures were prepared. Standard mixture 1 contained all hydrophilic phycotoxins except PSP toxins, at a concentration of 1 µg mL^−1^ in water: TTX, anhTTX (117 ng mL^−1^), BMAA, DAB, DA, ATX, and CYN. Standard mixture 2 contained all PSP toxins at a concentration of 1 µg mL^−1^ in water containing 0.03 M acetic acid: STX, NEO, dcSTX, dcNEO, GTX1 and 4 (GTX4 325 ng mL^−1^), GTX2 and 3 (GTX3 380 ng mL^−1^), GTX5, dcGTX2 and 3 (dcGTX3 224 ng mL^−1^), and C1 and 2 (C2 299 ng mL^−1^). Standard mixture 3 contained all microcystins at a concentration of 1 µg mL^−1^ in methanol/water (80:20 *v*/*v*): MC-LA, MC-LF, MC-LR, MC-LW, MC-LY, MC-RR, MC-WR, MC-HilR, MC-HtyR, MC-YR, dmMC-LR, and NOD. Standard mixture 4 contained lipophilic toxins at a concentration of 100 ng mL^−1^ in methanol: OA, DTX1, DTX2, YTX, hYTX, PTX2, SPX1, GYM, 13,19-didesMeSPXC, 20MeSPXG, 16:0 OA ester, OA C8-diol ester, AZA1, AZA2, AZA3, and AZA4. Standard mixture 5 contained all other lipophilic phycotoxins at a concentration of 100 ng mL^−1^ in methanol: PnTX E, PnTX F, PnTX G, pCTX1 (50 ng mL^−1^), pCTX2 (50 ng mL^−1^), pCTX3 (50 ng mL^−1^), PbTx2, PbTx3, PbTx9, PlTX, AZA5, OA methyl ester, and DA.

For the validation, some phycotoxins were excluded due to lack of standards (pCTX1, 2 and 3), poor sensitivity (PlTX), or poor peak shapes (DAB and BMAA) during method development. For the validation, mixtures 4 and 5 were combined.

Reference materials used during validation were CRM-ASP-mus-d and CRM-FDMT-1; these were purchased from NRC CNRC, Halifax, Canada. The CRM-FDMT-1 material was reconstituted according to the instructions. PO PST CRM 1101 was purchased from Centre for Environment, Fisheries, and Aquaculture Science (Cefas), Weymouth, United Kingdom.

### 4.2. Preparation of Extracts

#### 4.2.1. Fish and Shellfish Tissue Samples

The extraction procedure for fish and shellfish tissue was as follows: 1.0 ± 0.05 g tissue homogenate was weighed and extracted with 4 mL methanol. The sample was vortex-mixed for one minute using a multi-pulse vortex. The extract was centrifuged at 2000× *g* for 5 min, and the supernatant was decanted from the pellet to a graduated tube. A total of 5 mL of H_2_O/ACN/Amm.form/FA (55:45 *v*/*v*, 2 mM, 0.5 mM) was added to the pellet. The extract was again vortex-mixed for one minute using a multi-pulse vortex. The extract was centrifuged at 2000× *g* for 5 min, and the supernatant was combined with the previously obtained methanol extract. The tube was filled up to 10 mL with acetonitrile. To avoid the loss of compounds, two different filters suitable for lipophilic and hydrophilic phycotoxins were used. For the analysis of lipophilic phycotoxins, an aliquot of the extract was filtered with a 0.2 µm HT Tuffryn filter (Sigma-Aldrich, Zwijndrecht, The Netherlands). For the analysis of hydrophilic phycotoxins, an aliquot of the extract was filtered with a 0.45 µm PVDF filter (Sigma-Aldrich, Zwijndrecht, The Netherlands). The filtered extracts were transferred into a glass vial and used for analysis with LC-HRMS.

#### 4.2.2. Water Samples

The clean-up procedures for water samples were as follows: For the clean-up of water containing lipophilic phycotoxins, a 30 mg Strata-X polymeric reversed phase cartridge (Phenomenex, Utrecht, The Netherlands) was used. The cartridge was activated and conditioned with 1 mL methanol, followed by 1 mL water. A water sample of 1 mL was loaded into the cartridge, and the cartridge was washed with 1 mL water. Subsequently, the lipophilic phycotoxins were eluted with 1 mL methanol. The eluent was transferred into a glass vial and used for analysis with LC-HRMS. To extract hydrophilic phycotoxins from water, a 500 mg Chromabond HILIC cartridge (Macherey-Nagel, Düren, Germany) was used. The cartridge was activated and conditioned with 1 mL water, followed by 6 mL acetonitrile. A water sample of 1 mL diluted with 9 mL acetonitrile was loaded onto the cartridge and subsequently washed with 2 mL acetonitrile. The hydrophilic phycotoxins were eluted with 2 mL water. The eluent was diluted with 2 mL acetonitrile and transferred into a glass vial for analysis with LC-HRMS.

#### 4.2.3. Food Supplements

The extraction and clean-up procedure for food supplements was based on the methods developed for tissue and water. Pills were ground or capsules were removed beforehand. An amount of 1.0 ± 0.05 g of food supplement was weighed and extracted with 4 mL methanol. The extract was vortex-mixed for one minute using a multi-pulse vortex. Subsequently, the extract was ultrasonically disrupted for 1 min at 11 W (RMS), 40 kHz to disrupt possible intact algal cells. The extract was centrifuged at 2000× *g* for 5 min, and the supernatant was decanted from the pellet to a graduated tube. In total, 5 mL of H_2_O/ACN/Amm.form/FA (55:45 *v*/*v*, 2 mM, 0.5 mM) was added to the pellet. The extract was vortex-mixed for one minute using a multi-pulse vortex. The extract was centrifuged at 2000× *g* for 5 min, and the supernatant was combined with the methanol extract. The tube was filled to 10 mL with acetonitrile. The extract was diluted with 67.5 mL water in order to obtain 10% organic strength prior to the SPE procedure for lipophilic phycotoxins. A 60 mg Strata-X polymeric reversed phase cartridge was activated and conditioned with 3 mL methanol and 3 mL water. The diluted extract was loaded onto the cartridge and was washed with 3 mL water. The lipophilic phycotoxins were eluted with 2 mL methanol, and the eluate was transferred to a sample vial for analysis with LC-HRMS. A clean-up for hydrophilic phycotoxins in food supplements was not developed. A similar approach can be used for the hydrophilic toxins; however, as the method for lipophilic toxins did not give satisfactory results, and because an HILIC clean-up would be much more sensitive for the variety of matrices that were to be used as food supplements, it was decided not to test the approach on the hydrophilic toxins.

### 4.3. Liquid Chromatography–Mass Spectrometry (LC–OrbitrapMS)

#### 4.3.1. Reversed Phase Chromatography

For the screening of phycotoxins, a Thermo Scientific UltiMate 3000 LC-system (Thermo Fisher Scientific, Waltham, MS, USA) coupled with a Thermo Scientific Q Exactive focus hybrid quadrupole-orbitrap mass spectrometer was used. Mobile phase A consisted of water and mobile phase B consisted of acetonitrile/water (9:1 *v*/*v*), both solutions containing 2 mM ammonium formate and 0.5 mM formic acid. Chromatographic separation of lipophilic phycotoxins was achieved on a reversed phase ACQUITY BEH C_18_ 1.7 µm, 100 · 2.1 mm UPLC column (Waters, Milford, MA, USA). The column temperature was set at 35 °C, and the total run time was 28 min. The gradient elution with a flow of 0.3 mL min^−1^ was as follows: 0.1 min at 10% mobile phase B, then linearly increased to 100% mobile phase B for 12.9 min, and kept at 100% mobile phase B for 12 min. Subsequently, the gradient went back to 10% mobile phase B for 0.1 min, and then kept at 10% mobile phase B for 2.9 min to equilibrate the column for the next run.

#### 4.3.2. HILIC Chromatography

Chromatographic separation of hydrophilic phycotoxins was achieved on a TSK gel Amide-80 2 µm, 150 · 3 mm HPLC column (Tosoh Bioscience, Tokyo, Japan). The column temperature was set at 35 °C, and the total run time was 20 min. The gradient elution with a flow of 0.5 mL min^−1^ was as follows: 0.1 min at 90% mobile phase B, then linearly decreased to 45% mobile phase B for 13.9 min, and subsequently linearly decreased to 20% mobile phase B for 0.1 min, and then kept at 20% mobile phase B for 1.9 min. Subsequently, the gradient went back to 90% mobile phase B for 0.1 min and was kept at 90% mobile phase B for 3.9 min to equilibrate the column. For both chromatographic methods, the injection volume was set at 10 µL.

#### 4.3.3. MS Setting

In order to detect the phycotoxins, electrospray ionization (ESI) in both positive and negative modes was used. The positive and negative ESI signals were acquired in two separate runs. The spray voltage in the positive ionization mode was set at 3.5 kV and in the negative ionization mode at −2.5 kV. The capillary temperature was set at 260 °C. A full MS scan event of 100–1500 *m*/*z* with a resolution of 70,000 full width at half maximum (FWHM) was acquired. In order to obtain additional information on the phycotoxins, fragmentation spectra were also acquired. The so-called MS2 scans were obtained by selecting all ions in respective *m*/*z* mass range windows of 100–500, 500–1000, and 1000–1500. Nitrogen was used as collision gas. The normalized collision energy (NCE) was set at 40 during the fragmentation of all ion mass ranges, except for 100–500 *m*/*z* in the negative ionization mode where the NCE was set at 30. Then, after fragmentation, the ions were scanned respectively from 50 to 500, from 50 to 1000, and from 50 to 1500 *m*/*z,* with the resolution set at 17,500 FWHM. The automatic gain control representing the maximum capacity of the C-trap was set at a maximum of 10^6^ ions, or a maximum injection time of 200 ms for both the full scan and MS2 scans was allowed.

### 4.4. Data Processing

Tracefinder (Thermo Fisher Scientific, Waltham, MA, USA) was used for data processing. A database containing over 1100 phycotoxins was constructed from the literature (S2: phycotoxin database). The database contains information about molecular formulas, CAS numbers, availability of standards, indicative retention times, MS adducts and fragments, symptoms, and origin of the toxin. Relevant parts of the database for mass spectrometric data processing were transferred to a Tracefinder compound database (Thermo Fisher Scientific, Waltham, MA, USA). The raw data were processed with the Tracefinder software. A mass error of 5 parts per million (ppm) for the *m*/*z* of the precursor ion was allowed, and for the available phycotoxins, at least one fragment ion should also be present within a 5 ppm mass error. Furthermore, the retention time of the peak in the sample should be within a ±0.2 min retention time window of the retention time observed in the available standard. The retention time criteria applies to both the precursor and the fragment ions.

### 4.5. Validation

The following phycotoxins were validated: OA, DTX1, DTX2, YTX, hYTX, PTX2, SPX1, GYM, 13,19 didesm SPX C, 20 meth SPX G, OA methyl ester, 16:0 OA ester, OA C_8_ diol ester, AZA1, AZA2, AZA3, AZA4, AZA5, PnTX E, PnTX F, PnTX G, CYN, DA, MC-LA, MC-LF, MC-LR, MC-LW, MC-LY, MC-RR, MC-WR, MC-HilR, MC-HtyR, MC-YR, Asp MC-LR, NOD, STX, dcSTX, NEO, dcNEO, GTX1 and 4, GTX2 and 3, GTX5, dcGTX2 and 3, C1 and 2, TTX, and ATX. The validation of the screening method was based on the estimated screening detection limit (SDL). The SDL of the qualitative screening method is the lowest level at which an analyte is detected in at least 95% of the samples. A total of 20 blank tissue samples, including 4 mussel-, 4 oyster-, 4 cockle-, 4 ensis-, and a pangasius, salmon, mackerel, and shrimp homogenate were spiked after extraction with 600 µg kg^−1^ hydrophilic phycotoxins, 150 µg kg^−1^ microcystins, and 80 µg kg^−1^ of all other lipophilic phycotoxins included in the validation. Five blanks, one from each matrix, were included to determine false positives. A total of 20 blank water samples, including 6 sea water, 6 brackish water, 6 fresh water, and 2 tap water samples were spiked before clean-up with 120 µg L^−1^ hydrophilic phycotoxins, 10 µg L^−1^ MCs, and 5 µg L^−1^ of all other lipophilic phycotoxins. DA was included in both clean-ups for hydrophilic phycotoxins and lipophilic phycotoxins. Furthermore, 5 blank water samples were included to determine false positives. In total, 20 blank solid food supplements (with a nature of algae or (shell)fish were spiked before extraction with 30 µg kg^−1^ MCs and 15 µg kg^−1^ of all other lipophilic phycotoxin standards. There were 20 blank liquid food supplements (mostly oils) that were spiked before extraction with 50 µg kg^−1^ MCs and 15 µg kg^−1^ of all other lipophilic phycotoxins. A total of 9 blank food supplements were included to determine false positives. For each procedure, an empty tube was also included to determine if there were any interfering contaminants from the procedures themselves. Furthermore, the confirmation and quantitation of regulated lipophilic phycotoxins and some Cis, PSP toxins, and DA (OA, DTX1, DTX2, YTX, hYTX, AZA1, AZA2, AZA3, SPX1, GYM, PnTX G, STX, dcSTX, NEO, dcNEO, GTX1 and 4, GTX2 and 3, GTX5, dcGTX2 and 3 and DA) in shellfish were validated. The correlation coefficient should be greater than 0.99, and the back-calculated concentration of the matrix-matched standards should not deviate by more than 20% of the theoretical concentration. The drift between two bracketing calibration curves should not exceed a 30% difference in the slopes. Five blank mussel homogenates were spiked after extraction at 0.5 and 1 times the target value. One blank mussel homogenate was spiked before extraction at 0.5 times the target value- and reference materials were included to determine the recovery. Matrix matched standards were spiked at 5 (ASP and PSP) or 6 (lipophilic phycotoxins) different concentration levels. Concentrations are shown in Table 2.

## Figures and Tables

**Table 1:** Average recoveries (%) (*n* = 2) of lipophilic phycotoxins in food supplements. Recovery is based on spikes before and after extraction.

Sample	OA	DTX1	DTX2	AZA1	AZA2	AZA3	AZA4	YTX	hYTX	SPX1	GYM	PTX2	Average
Oil	80	80	82	59	46	70	89	62	56	94	92	91	75
Oil caps.	56	67	66	27	22	39	84	13	9	91	84	64	52
Powder	91	61	79	49	48	53	57	25	27	62	74	54	57
Pills	78	82	79	35	24	46	69	20	18	79	80	80	76
Average	76	73	77	43	35	52	75	30	27	81	82	72	
MC-	LR	RR	WR	HtyR	YR	LW	HilR	LY	LF	dmLR	LA	NOD	
Oil	86	76.	84	79	75	81	84	84	90	82	73	84	81
Oil caps.	46	102	66	56	55	20	54	15	18	50	10	41	44
Powder	57	84.	53	57	53	69	62	54	56	58	53	67	60
Pills	60	50	54	62	65	63	64	45	50	65	44	63	57
Average	62	78	64	64	6	58	66	49	53	64	44	64	

**Table 2:** Spike levels of ASP, PSP and lipophilic toxins for quantitative validation in shellfish.

Compound	Toxin Group	0.5× (µg kg^−1^)	1× (µg kg^−1^)	Matrix-Matched Standards (µg kg^−1^)
DA	ASP	10,000	20,000	0, 5000, 10,000, 20,000, 50,000
STX, dcSTX, NEO, dcNEO, GTX1 and 4 ^1^, GTX2 and 3 ^1^, GTX5, dcGTX2 and 3 ^1^	PSP	400	800	0, 400, 600, 800, 1200
OA, DTX1, DTX2	DSP	80	160	0, 20, 40, 80, 160, 240
AZA1, AZA2, AZA3		80	160	0, 20, 40, 80, 160, 240
YTX, hYTX		250	500	0, 62.5, 125, 250, 500, 750
SPX1		200	400	0, 50, 100, 200, 400, 600
GYM		100	200	0, 25, 50, 100, 200, 300
PnTX G		25	50	0, 6.25, 12.5, 25, 50, 75

^1^ Concentration of the highest isomer present in the standard mix.

## Data Availability

The data presented in this study are available in Supplementary Materials S2 and S3.

## Supplementary Materials

The following are available online at https://www.mdpi.com/article/10.3390/toxins13110823/s1, S1: structures; S2: phycotoxin database; S3: Validation data, Tables S3.1–S3.13: validation data, Tables S4.1–S4.2: standards.
