# Supplementary material for: A Generic LC-HRMS Screening Method for Marine and Freshwater Phycotoxins in Fish, Shellfish, Water, and Supplements
Source: Toxins (Basel). 2021 Nov 22;13(11):823. doi: 10.3390/toxins13110823 (PMC8619867; doi:10.3390/toxins13110823)
Supplement: Supplementary file 1 [file toxins-13-00823-s001.zip › toxins-1443507-su-11.22.pdf]

toxins MDPI

Supplementary Materials: A generic LC-HRMS screening method for marine and freshwater phycotoxins in fish, shellfish, water and supplements

Mirjam D. Klijnstra, Elisabeth J. Faassen and Arjen Gerssen

S1: Structures

β-N-methylamino-L-alanine

Abbreviation: BMAA
Cas number: 15920-93-1
Molecular formula: $C_4H_{10}N_2O_2$
Exact mass: 118.074228
Group: Hydrophilic

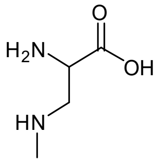

D-2,4-Diaminobutyric acid

Abbreviation: DAB
Cas number: 26908-94-1
Molecular formula: $C_4H_{10}N_2O_2$
Exact mass: 118.074228
Group: Hydrophilic

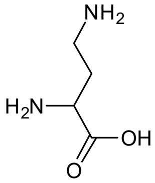

Anatoxin-a

Abbreviation: ATX
Cas number: 64285-06-9
Molecular formula: $C_{10}H_{15}NO$
Exact mass: 165.115364
Group: Hydrophilic

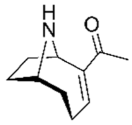

Domoic acid

Abbreviation: DA
Cas number: 14277-97-5
Molecular formula: $C_{15}H_{21}NO_6$
Exact mass: 311.136889
Group: ASP, Hydrophilic

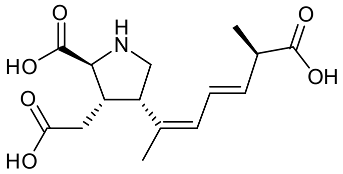

Tetrodotoxin

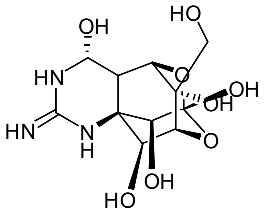

Abbreviation: TTX
Cas number: 4368-28-9
Molecular formula: $C_{11}H_{17}N_3O_8$
Exact mass: 319.101567
Group: Hydrophilic

PSP toxins, hydrophilic

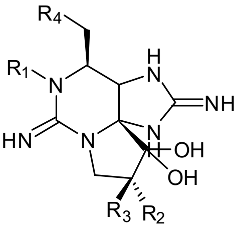

Group: Hydrophilic

Name	Abbr.	Cas number	Molecular formula	Exact mass	R1	R2	R3	R4
Saxitoxin	STX	35523-89-8	$C_{10}H_{17}N_7O_4$	299.134203	H	H	H	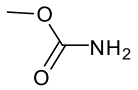
Neosaxitoxin	NEO	64296-20-4	$C_{10}H_{17}N_7O_5$	315.129118	OH	H	H	
Gonyautoxin 1	GTX1	60748-39-2	$C_{10}H_{17}N_7O_9S$	411.080850	OH	H	OSO_3^-	
Gonyautoxin 2	GTX2	60508-89-6	$C_{10}H_{17}N_7O_8S$	395.085935	H	H	OSO_3^-	
Gonyautoxin 3	GTX3	60537-65-7	$C_{10}H_{17}N_7O_8S$	395.085935	H	OSO_3^-	H	
Gonyautoxin 4	GTX4	64296-26-0	$C_{10}H_{17}N_7O_9S$	411.080850	OH	OSO_3^-	H	
Gonyautoxin 5	GTX5	64296-25-9	$C_{10}H_{17}N_7O_7S$	379.091020	H	H	H	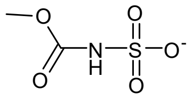
N-sulfocarbamoyl gonyautoxin 2	C1	80173-30-4	$C_{10}H_{17}N_7O_{11}S_2$	475.042752	H	H	OSO_3^-	
N-sulfocarbamoyl gonyautoxin 3	C2	80226-62-6	$C_{10}H_{17}N_7O_{11}S_2$	475.042752	H	OSO_3^-	H	
Decarbamoylsaxitoxin	dcSTX	58911-04-9	$C_9H_{16}N_6O_3$	256.128389	H	H	H	OH
Decarbamoylneosaxitoxin	dcNEO	68683-58-9	$C_9H_{16}N_6O_4$	272.123304	OH	H	H	OH
Decarbamoylgonyautoxin 2	dcGTX2	86996-87-4	$C_9H_{16}N_6O_7S$	352.080121	H	H	OSO_3^-	OH
Decarbamoylgonyautoxin 3	dcGTX3	87038-53-7	$C_9H_{16}N_6O_7S$	352.080121	H	OSO_3^-	H	OH

Cylindrospermopsin

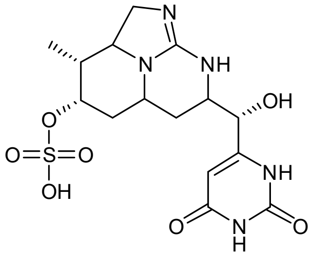

Abbreviation: CYN
Cas number: 143545-90-8
Molecular formula: $C_{15}H_{21}N_5O_7S$
Exact mass: 415.116172
Group: Hydrophilic

Gymnodimine A

Abbreviation: GYM A
Cas number: 173792-58-0
Molecular formula: $C_{32}H_{45}NO_4$
Exact mass: 507.334859
Group: Cyclic imines, Lipophilic

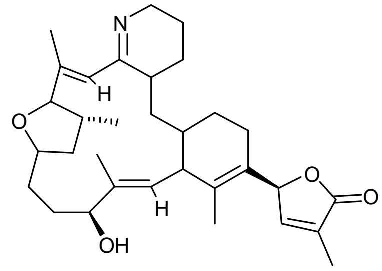

Spirolide C

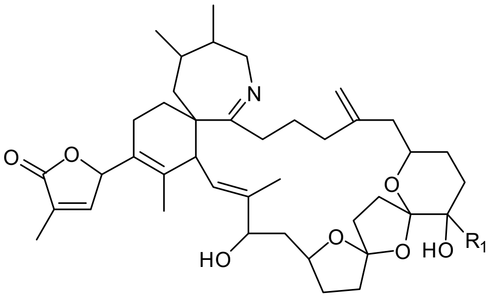

Group: Cyclic imines, Lipophilic

Name	Abbr.	Cas number	Molecular formula	Exact mass	R1
13-Desmethyl spirolide C	SPX1	334974-07-1	$C_{42}H_{61}NO_7$	691.444804	CH_3
13,19-Didesmethyl spirolide C	13,19-didesMeSPXC	908118-02-5	$C_{41}H_{59}NO_7$	677.429154	H

20-Methyl spirolide G

Abbreviation: 20MeSPXG
Cas number: 849215-95-8
Molecular formula: $C_{43}H_{63}NO_7$
Exact mass: 705.460454
Group: Cyclic imines, Lipophilic

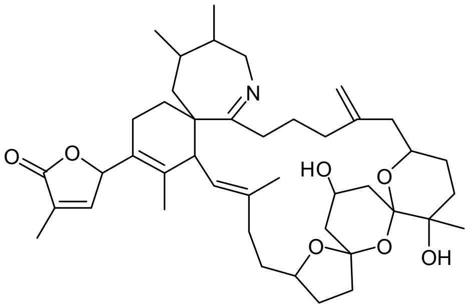

Pinnatoxins

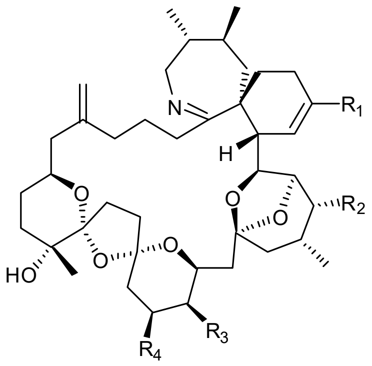

Group: Cyclic imines, Lipophilic

Name	Abbr.	Cas number	Molecular formula	Exact mass	R1	R2	R3	R4
Pinnatoxin E	PnTX E	1227167-69-2	$C_{45}H_{69}NO_{10}$	783.492149	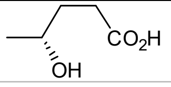	H	OH	CH_3
Pinnatoxin F	PnTX F	1227167-70-5	$C_{45}H_{67}NO_9$	765.481584	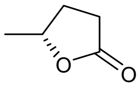	H	OH	CH_3
Pinnatoxin G	PnTX G	1312711-74-2	$C_{42}H_{63}NO_7$	693.460454	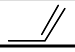	OH	H	H

Azaspiracids

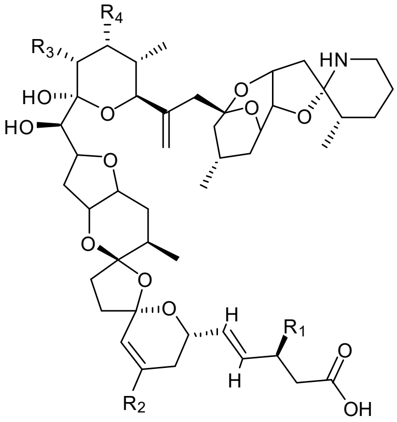

Group: Lipophilic

Name	Abbr.	Cas number	Molecular formula	Exact mass	R1	R2	R3	R4
Azaspiracid 1	AZA1	214899-21-5	$C_{47}H_{71}NO_{12}$	841.497629	H	H	CH_3	H
Azaspiracid 2	AZA2	265996-92-7	$C_{48}H_{73}NO_{12}$	855.513279	H	CH_3	CH_3	H
Azaspiracid 3	AZA3	265996-93-8	$C_{46}H_{69}NO_{12}$	827.481979	H	H	H	H
Azaspiracid 4	AZA4	344422-49-7	$C_{46}H_{69}NO_{13}$	843.476894	OH	H	H	H
Azaspiracid 5	AZA5	344422-51-1	$C_{46}H_{69}NO_{13}$	843.476894	H	H	H	OH

Pectenotoxin 2

Abbreviation: PTX2

Cas number: 97564-91-5

Molecular formula: $C_{47}H_{70}O_{14}$

Exact mass: 858.476560

Group: Lipophilic

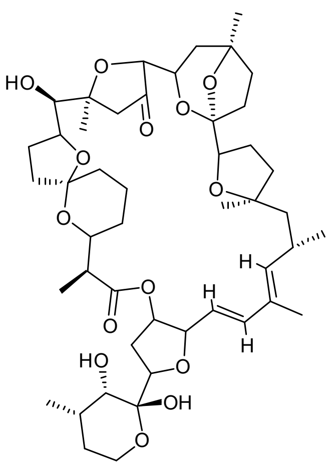

Nodularin

– D-MeAsp – L-Arg – Adda – D-Glu – Mdhb –

Abbreviation: NOD

Cas number: 118399-22-7

Molecular formula: $C_{41}H_{60}N_8O_{10}$

Exact mass: 824.443242

Group: Lipophilic

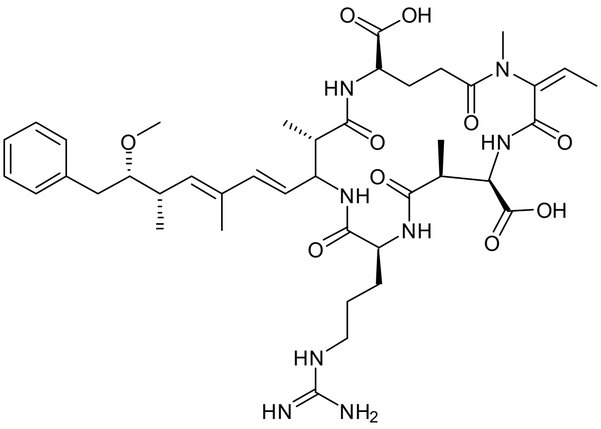

Microcystins

– D-Ala – X –D- MeAsp – Y – Adda – D-Glu – Mdha –

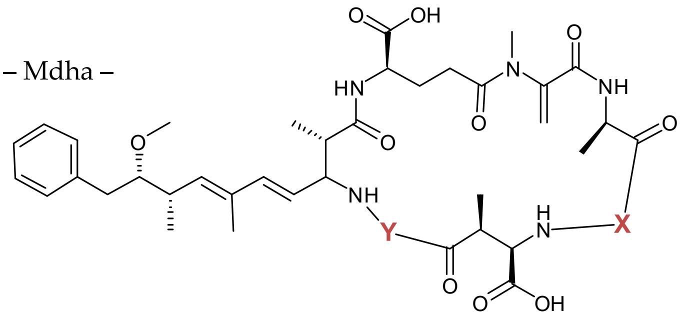

Group: Lipophilic

Name	Abbr.	Cas number	Molecular formula	Exact mass	X	Y
Microcystin HilR	MC-HilR	169789-55-3	$C_{50}H_{76}N_{10}O_{12}$	1008.56442	Hil	Arg
Microcystin HtyR	MC-HtyR	478001-08-0	$C_{53}H_{74}N_{10}O_{13}$	1058.543685	Hty	Arg
Microcystin LA	MC-LA	96180-79-9	$C_{46}H_{67}N_7O_{12}$	909.484773	Leu	Ala
Microcystin LF	MC-LF	154037-70-4	$C_{52}H_{71}N_7O_{12}$	985.516073	Leu	Phe
Microcystin LR	MC-LR	101043-37-2	$C_{49}H_{74}N_{10}O_{12}$	994.548770	Leu	Arg
Microcystin LW	MC-LW	157622-02-1	$C_{54}H_{72}N_8O_{12}$	1024.526972	Leu	Trp
Microcystin LY	MC-LY	123304-10-9	$C_{52}H_{71}N_7O_{13}$	1001.510988	Leu	Tyr
Microcystin RR	MC-RR	111755-37-4	$C_{49}H_{75}N_{13}O_{12}$	1037.565817	Arg	Arg
Microcystin WR	MC-WR	138234-58-9	$C_{54}H_{73}N_{11}O_{12}$	1067.544019	Trp	Arg
Microcystin YR	MC-YR	101064-48-6	$C_{52}H_{72}N_{10}O_{13}$	1044.528035	Tyr	Arg
Asp^3 microcystin LR	AspMC-LR	120011-66-7	$C_{48}H_{72}N_{10}O_{12}$	980.533120	Leu	Arg

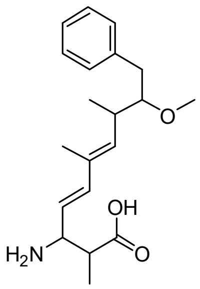

Adda

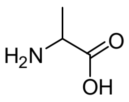

Ala

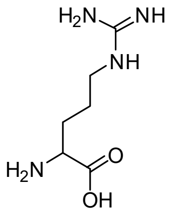

Arg

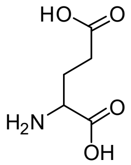

Glu

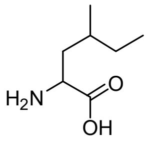

Hil

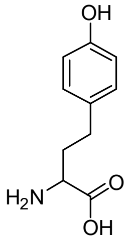

Hty

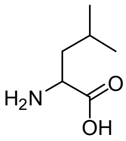

Leu

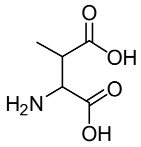

MeAsp

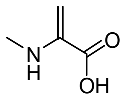

Mdha

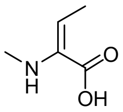

Mdhb

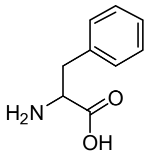

Phe

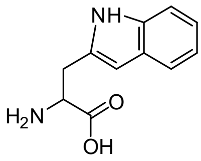

Trp

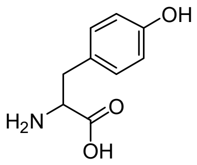

Tyr

DSP

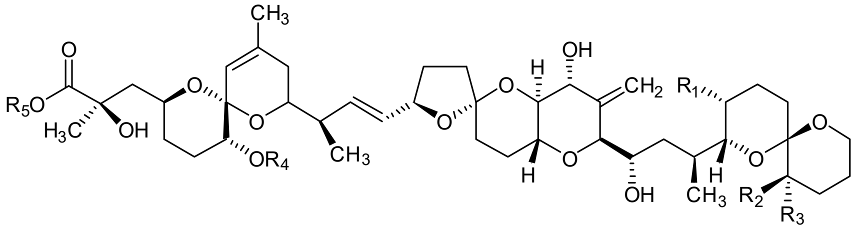

Group: Lipophilic

Name	Abbr.	Cas number	Molecular formula	Exact mass	R1	R2	R3	R4	R5
Okadaic acid	OA	78111-17-8	$C_{44}H_{68}O_{13}$	804.465995	CH_3	H	H	H	H
Dinophysistoxin 1	DTX1	81720-10-7	$C_{45}H_{70}O_{13}$	818.481645	CH_3	CH_3	H	H	H
Dinophysistoxin 2	DTX2	139933-46-3	$C_{44}H_{68}O_{13}$	804.465995	H	H	CH_3	H	H
16:0 7-O-Acyl okadaic acid	DTX3	118745-19-0	$C_{60}H_{98}O_{14}$	1042.695660	CH_3	H	H	$(CH_2)_{14}CH_3$	H
Okadaic acid methyl ester	OA methyl ester	78111-14-5	$C_{45}H_{70}O_{13}$	818.481645	CH_3	H	H	H	CH_3
Okadaic acid-D8a	OA C_8-diol ester	318536-96-8	$C_{52}H_{80}O_{14}$	928.554810	CH_3	H	H	H	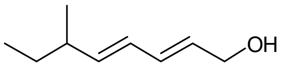

Brevetoxins

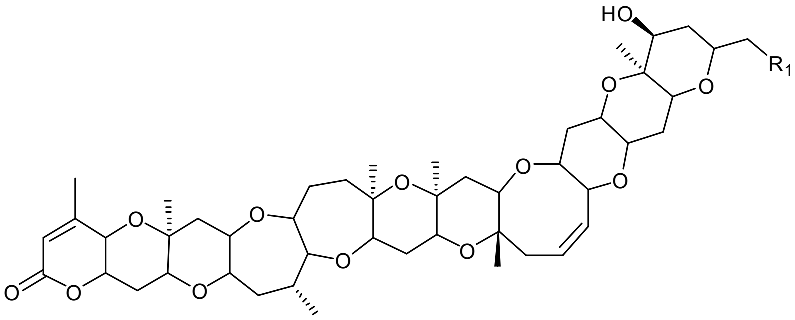

Group: Lipophilic

Name	Abbr.	Cas number	Molecular formula	Exact mass	R1
Brevetoxin 2	PbTx 2	79580-28-2	C50H70O14	894.476560	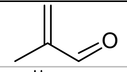
Brevetoxin 3	PbTx 3	85079-48-7	C50H72O14	896.492210	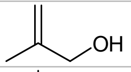
Brevetoxin 9	PbTx 9	142353-09-1	C50H74O14	898.507860	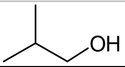

Yessotoxins

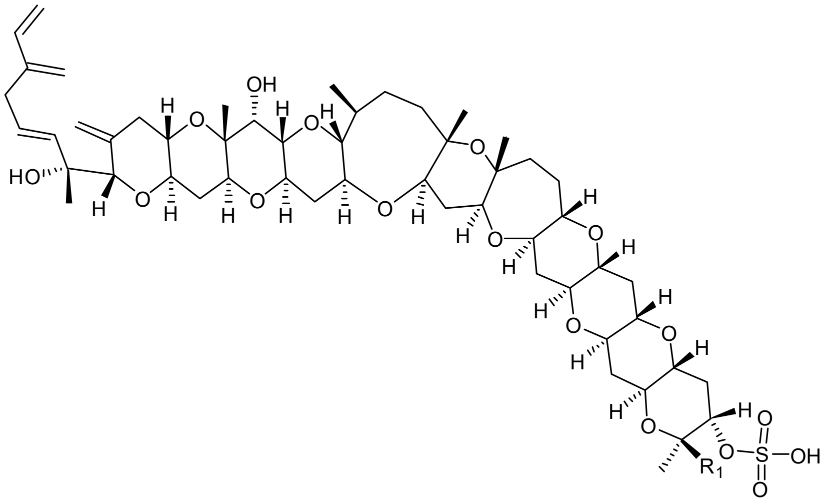

Group: Lipophilic

Name	Abbr.	Cas number	Molecular formula	Exact mass	R1
Yessotoxin	YTX	112514-54-2	$C_{55}H_{82}O_{21}S_2$	1142.479009	$(CH_2)_2OSO_3^-$
1a-Homoyessotoxin	hYTX	196309-94-1	$C_{56}H_{84}O_{21}S_2$	1156.494659	$(CH_2)_3OSO_3^-$

Pacific ciguatoxins

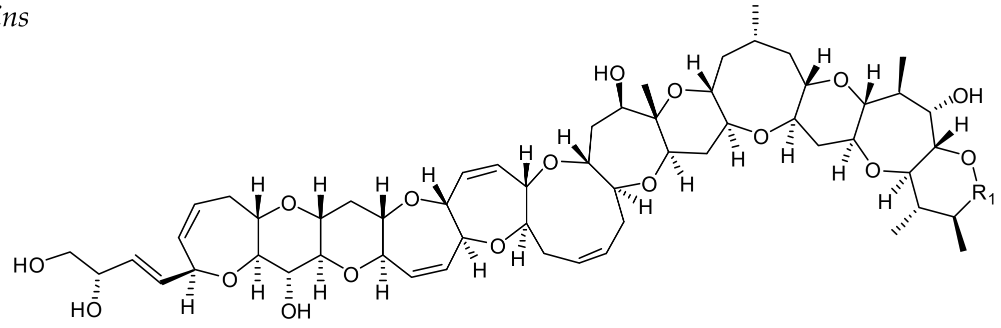

Group: Lipophilic

Name	Abbr.	Cas number	Molecular formula	Exact mass	R1
Pacific ciguatoxin 1	P-CTX-1	11050-21-8	$C_{60}H_{86}O_{19}$	1110.576335	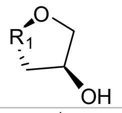
Pacific ciguatoxin 2	P-CTX-2	142185-85-1	$C_{60}H_{86}O_{18}$	1094.581420	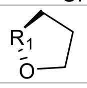
Pacific ciguatoxin 3	P-CTX-3	139341-09-6	$C_{60}H_{86}O_{18}$	1094.581420	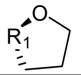

Palytoxin

Abbreviation:	PlTX
Cas number:	77734-92-0
Molecular formula:	$C_{129}H_{223}N_3O_{54}$
Exact mass:	2678.479607
Group:	Lipophilic

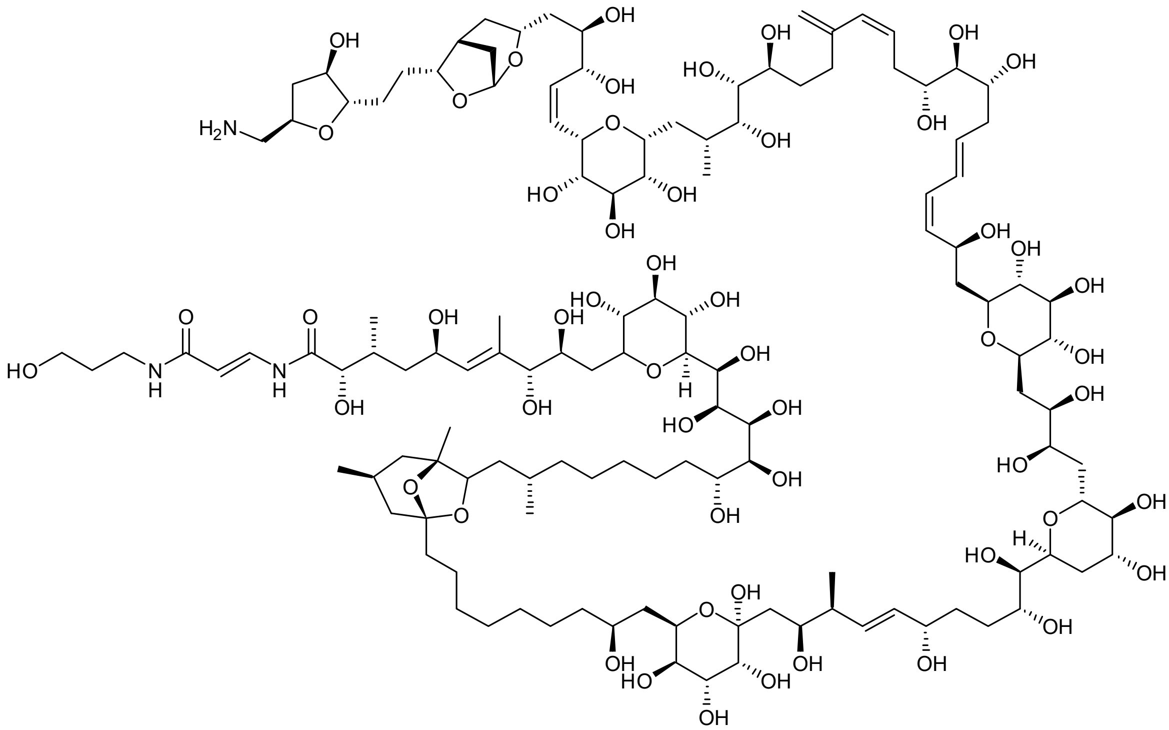

S3: Validation data

Screening of hydrophilic and lipophilic phycotoxins in shellfish and fish

Spiking levels: Hydrophilic phycotoxins 600 µg kg^{-1}*

Microcystins and nodularin 150 µg kg^{-1}

Other lipophilic phycotoxins80 µg kg^{-1}

*Exceptions: dcGTX3 (134 µg kg^{-1}), GTX1&4 (795 µg kg^{-1}), GTX3 (228 µg kg^{-1}), C2 (180 µg kg^{-1})

Table S3.1. Validation results of hydrophilic phycotoxins in shellfish and fish.

**HILIC POS**	**S**	**blanks**						**mussel**				**cockle**				**oyster**				**ensis**				**fish**				**S**
ATX	3	1	0	1	0	0	1	2	3	2	2	2	3	3	3	3	2	2	3	2	2	3	3	3	3	3	3	3
dcNEO	3	0	0	0	0	0	0	3	2	1	3	3	3	3	2	3	3	3	3	2	2	3	3	3	3	2	3	3
dcSTX	3	0	0	0	0	0	0	3	3	2	3	3	3	3	3	3	3	3	3	3	3	3	3	3	3	3	3	3
NEO	3	0	0	0	0	0	0	3	3	1	3	3	3	3	2	3	3	3	3	3	2	3	3	3	3	2	3	3
STX	3	0	0	0	0	0	0	3	3	2	3	3	3	3	3	3	3	3	3	3	3	3	3	3	3	3	3	3
TTX	3	0	0	0	0	0	0	3	3	2	2	2	3	2	3	3	2	2	3	1	1	2	3	3	2	2	3	3
**HILIC NEG**																												
CYN	2	0	0	0	0	0	0	2	2	2	2	2	2	1	2	2	2	2	2	1	2	2	1	2	2	2	2	2
dcGTX2	3	0	1	0	0	0	0	3	3	3	3	3	3	3	3	3	3	3	3	3	3	3	3	3	3	3	3	3
dcGTX3	3	1	0	0	0	0	0	3	3	2	2	2	3	3	3	3	3	3	3	3	2	3	3	3	3	3	3	3
DA	2	0	1	1	0	0	0	2	2	2	2	2	2	2	2	2	2	2	2	2	2	2	2	2	2	2	2	2
GTX1&4	3	0	0	0	0	0	1	2	3	3	3	3	3	3	3	2	3	2	2	3	2	2	3	3	3	3	3	3
GTX2	3	0	0	0	0	0	0	3	3	3	3	3	3	3	3	3	3	3	3	3	3	3	2	3	3	3	3	3
GTX3	3	0	0	0	0	0	0	3	3	3	3	3	3	3	3	3	3	3	3	2	2	3	3	3	3	2	3	3
GTX5	3	0	0	0	0	1	0	3	3	3	3	3	3	3	3	3	3	2	2	2	2	2	2	3	3	3	3	3
C1	3	0	0	0	0	0	0	1	1	3	2	2	2	2	2	2	2	2	3	2	2	2	3	3	2	3	3	3
C2	2	0	0	1	0	0	0	2	2	2	2	2	1	2	2	1	1	1	2	1	1	2	2	2	2	2	2	2

**LEGEND**	
0	No precursor ion found
1	Precursor ion found
2	Precursor ion + 1 fragment ion found
3	Precursor ion + 2 fragment ions found
S	Standard solution
HILIC POS	Measurements with HILIC and positive electrospray ionization
HILIC NEG	Measurements with HILIC and negative electrospray ionization

Table S3.2. Validation results of lipophilic phycotoxins in shellfish and fish.

RP POS	S	blanks						mussel				cockle				oyster				ensis				fish				S
MC-HilR	3	0	0	0	0	0	0	3	3	3	3	3	3	3	3	3	3	3	3	3	3	3	3	3	3	3	3	3
MC-HtyR	3	0	0	0	0	0	0	3	3	3	3	3	3	3	3	3	3	3	3	3	3	3	3	3	3	3	3	3
MC-LA	3	0	0	0	0	0	0	3	3	3	3	3	3	3	3	3	3	3	3	3	3	3	3	3	3	3	3	3
MC-LF	3	0	0	0	0	0	0	3	3	3	3	3	3	3	3	3	3	3	3	3	3	3	3	3	3	3	3	3
MC-LR	3	0	0	0	0	1	0	3	3	3	3	3	3	3	3	3	3	3	3	3	3	3	3	3	3	3	3	3
MC-LW	3	0	0	0	0	0	0	3	3	3	3	3	3	3	3	3	3	3	3	3	3	3	3	3	3	3	3	3
MC-LY	3	0	0	0	0	0	0	3	3	3	3	3	3	3	3	3	3	3	3	3	3	3	3	3	3	3	3	3
MC-RR	3	0	0	0	0	0	0	3	3	3	3	3	3	3	3	3	3	3	3	3	3	3	3	3	3	3	3	3
MC-WR	3	0	0	0	0	0	0	3	3	3	3	3	3	3	3	3	3	3	3	3	3	3	3	3	3	3	3	3
MC-YR	3	0	0	0	0	0	0	3	3	3	3	3	3	3	3	3	3	3	3	3	3	3	3	3	3	3	3	3
NOD	3	0	0	0	0	0	0	3	3	3	3	3	3	3	3	3	3	3	3	3	3	3	3	3	3	3	3	3
13,19-didesMeSPXC	3	0	0	0	0	0	0	3	3	3	3	3	3	3	3	3	3	3	3	3	3	3	3	3	3	3	3	3
SPX1	3	0	0	3	0	0	0	3	3	3	3	3	3	3	3	3	3	3	3	3	3	3	3	3	3	3	3	3
20MeSPXG	3	0	0	3	0	0	0	3	3	3	3	3	3	3	3	3	3	3	3	3	3	3	3	3	3	3	3	3
Asp MC-LR	3	0	0	0	0	0	0	3	3	3	3	3	3	3	3	3	3	3	3	3	3	3	3	3	3	3	3	3
AZA1	3	0	0	0	0	0	0	3	3	3	3	3	3	3	3	3	3	3	3	3	3	3	3	3	3	2	3	3
AZA2	3	0	0	0	0	0	0	3	3	3	3	3	3	3	3	3	3	3	3	3	3	3	3	3	3	3	3	3
AZA3	3	0	0	0	0	0	0	3	3	3	3	3	3	3	3	3	3	3	3	3	3	3	3	3	3	3	3	3
AZA4	3	0	0	0	0	0	0	3	3	3	3	3	3	3	3	3	3	3	3	3	3	3	3	3	3	3	3	3
AZA5	3	0	0	0	0	0	0	3	3	3	3	3	3	3	3	3	3	3	3	3	3	3	3	3	1	1	3	3
GYM	3	0	0	0	0	0	0	3	3	3	3	3	3	3	3	3	3	3	3	3	3	3	3	3	3	3	3	3
OA methyl ester	3	0	0	0	0	0	0	2	3	3	3	3	3	3	3	3	3	3	3	3	3	3	3	3	3	3	3	3
OA C_8-diol ester	3	0	0	0	0	0	1	3	3	3	3	3	3	3	3	3	3	3	3	3	3	3	2	3	3	2	3	3
PTX2	3	0	0	0	0	0	0	3	3	3	3	3	3	3	3	3	2	3	2	3	2	3	2	3	2	2	2	3
PnTX E	3	0	0	0	0	0	0	3	3	3	3	3	3	3	3	3	3	3	3	3	3	3	3	3	3	3	3	3
PnTX F	3	0	0	0	0	0	0	3	3	3	3	3	3	3	3	3	3	3	3	3	3	3	3	3	3	3	3	3
PnTX G	3	0	0	3	0	0	0	3	3	3	3	3	3	3	3	3	3	3	3	3	3	3	3	3	3	3	3	3
**RP NEG**																												
16:0 OA ester	1	0	0	0	0	0	0	1	1	1	1	1	1	1	1	1	1	1	1	1	1	1	1	1	1	1	1	1
hYTX	3	0	0	0	0	0	0	2	3	3	2	2	3	2	3	3	2	2	2	2	2	3	3	3	3	3	2	3
DTX1	3	0	0	0	0	0	0	3	3	3	3	3	3	3	3	3	3	3	3	3	3	3	3	3	3	3	3	3
DTX2	3	0	0	0	0	0	0	3	3	3	3	3	3	3	3	3	3	3	3	3	3	3	3	3	3	3	3	3
OA	3	0	0	0	0	0	0	3	3	3	3	3	3	3	3	3	3	3	3	3	3	3	3	3	3	3	3	3
YTX	3	0	0	0	0	0	0	0	2	2	3	3	0	2	3	2	2	2	0	2	2	2	2	3	2	2	3	3

LEGEND	
0	No precursor ion found
1	Precursor ion found
2	Precursor ion + 1 fragment ion found
3	Precursor ion + 2 fragment ions found
S	Standard solution
RP POS	Measurements with reversed phase LC and positive electrospray ionization
RP NEG	Measurements with reversed phase LC and negative electrospray ionization

Screening of hydrophilic and lipophilic phycotoxins in water

Spiking levels: Hydrophilic phycotoxins 120 µg L^{-1}*

Microcystins 10 µg L^{-1}

Other lipophilic phycotoxins5 µg L^{-1}

*Exceptions: dcGTX3 (27 µg L^{-1}), GTX1&4 (159 µg L^{-1}), GTX3 (46 µg L^{-1}), C2 (36 µg L^{-1})

Table S3.3. Validation results of hydrophilic phycotoxins in water.

HILIC POS	S			blanks					sea water						brackish water						fresh water			tap		S
ATX	3	0	0	0	0	0	2	2	2	2	3	2	2	2	2	3	2	2	3	2	3	3	2	2	3	3	3
dcNEO	3	0	0	0	0	0	3	2	3	2	2	3	2	3	3	3	3	3	3	3	3	3	3	3	3	3	3
dcSTX	3	0	0	0	0	0	3	3	3	1	2	3	3	3	3	3	3	3	3	3	3	3	3	3	3	3	3
NEO	3	0	0	0	0	0	3	1	3	1	2	3	3	3	3	3	3	3	3	3	3	3	3	3	3	3	3
STX	3	1	0	0	0	0	3	3	3	0	3	3	3	3	3	3	3	3	3	3	3	3	3	3	3	3	3
TTX	3	0	0	0	0	0	2	2	2	3	2	3	3	2	2	3	3	3	3	3	2	2	3	3	3	2	3
**HILIC NEG**																											
CYN	2	0	0	0	0	0	2	2	2	2	2	2	2	2	2	2	2	2	2	2	2	2	2	2	2	2	2
dcGTX2	3	0	0	0	0	0	3	3	3	3	1	3	3	3	3	3	3	3	3	2	3	3	3	3	3	3	3
dcGTX3	3	0	0	0	0	0	2	3	1	0	0	3	3	3	2	3	3	2	3	1	3	3	3	3	3	3	3
DA	2	0	0	0	0	0	0	2	0	2	0	0	1	0	0	0	0	1	0	0	1	2	1	1	0	0	2
GTX1&4	3	0	0	0	0	0	1	3	1	1	0	2	3	3	2	3	2	3	3	1	3	3	3	3	3	3	3
GTX2	3	0	0	0	0	0	3	3	3	1	2	3	3	3	3	3	3	3	3	3	3	3	3	3	3	3	3
GTX3	3	0	0	0	0	0	1	2	2	0	1	1	2	3	3	2	2	2	2	1	2	3	3	3	3	3	3
GTX5	3	0	0	0	0	0	0	3	0	3	0	0	3	2	2	2	2	2	3	2	3	3	3	3	3	3	3
C1	3	0	0	0	0	0	2	3	2	3	1	3	3	3	2	3	3	3	3	3	3	3	3	3	3	3	3
C2	2	0	0	0	0	0	1	1	2	2	0	1	2	1	1	1	2	1	2	1	2	2	2	2	2	2	2

LEGEND	
0	No precursor ion found
1	Precursor ion found
2	Precursor ion + 1 fragment ion found
3	Precursor ion + 2 fragment ions found
S	Standard solution
HILIC POS	Measurements with HILIC and positive electrospray ionization
HILIC NEG	Measurements with HILIC and negative electrospray ionization

Table S3.4. Validation results of lipophilic phycotoxins in water.

RP POS	S	blanks					sea water						brackish water						fresh water						tap		S
MC-HilR	3	0	0	0	0	0	3	3	3	3	3	3	3	3	3	3	3	3	3	3	3	3	3	3	3	3	3
MC-HtyR	3	0	0	0	0	0	3	3	3	3	3	3	3	3	3	3	3	3	3	3	3	3	3	3	3	3	3
MC-LA	3	0	0	0	0	0	3	3	3	3	3	3	3	3	3	3	3	3	3	3	3	3	3	3	3	3	3
MC-LF	3	0	0	0	0	0	3	3	3	3	3	3	3	3	3	3	3	3	3	3	3	2	3	3	3	3	3
MC-LR	3	0	0	0	0	0	3	3	3	3	3	3	3	3	3	3	3	3	3	3	3	3	3	3	3	3	3
MC-LW	3	0	0	0	0	0	3	3	3	3	3	3	3	3	3	3	3	3	3	3	3	3	3	3	3	3	3
MC-LY	3	0	0	0	0	0	3	3	3	3	3	3	3	3	3	3	3	3	3	3	3	3	3	3	3	3	3
MC-RR	3	0	0	0	0	0	3	3	3	3	3	3	3	3	3	3	3	3	3	3	3	3	3	3	3	3	3
MC-WR	3	0	0	0	0	0	3	3	3	3	3	3	3	3	3	3	3	3	3	3	3	3	3	3	3	3	3
MC-YR	3	0	0	0	0	0	3	3	3	3	3	3	3	3	3	3	3	3	3	3	3	3	3	3	3	3	3
NOD	3	0	0	0	0	0	3	3	3	3	3	3	3	3	3	3	3	3	3	3	3	3	3	3	3	3	3
13,19-didesMeSPXC	3	0	0	0	0	0	3	3	3	3	3	3	3	3	3	3	3	3	3	3	3	3	3	3	3	3	3
SPX1	3	0	0	3	0	0	3	3	3	3	3	3	3	3	3	3	3	3	3	3	3	3	3	3	3	3	3
20MeSPXG	3	0	0	0	0	0	3	3	3	3	3	3	3	3	3	3	3	3	3	3	3	3	3	3	3	3	3
Asp MC-LR	3	0	0	0	0	0	3	3	3	3	3	3	3	3	3	3	3	3	3	3	3	3	3	3	3	3	3
AZA1	3	0	0	0	0	0	3	3	3	3	3	3	3	3	3	3	3	3	3	3	3	3	3	3	3	3	3
AZA2	3	0	0	0	0	0	3	3	3	3	3	3	3	3	3	3	3	3	3	3	3	3	3	3	3	3	3
AZA3	3	0	0	0	0	0	3	3	3	3	3	3	3	3	3	3	3	3	3	3	3	3	3	3	3	3	3
AZA4	3	0	0	0	0	0	3	3	3	3	3	3	3	3	3	3	3	3	3	3	3	3	3	3	3	3	3
AZA5	3	0	0	0	0	0	3	3	3	3	3	3	3	3	3	3	3	3	3	3	3	3	3	3	3	3	3
GYM	3	0	0	2	0	0	3	3	3	3	3	3	3	3	3	3	3	3	3	3	3	3	3	3	3	3	3
OA methyl ester	2	0	0	0	0	0	2	2	2	2	2	2	2	2	3	3	2	2	2	3	3	2	2	3	3	3	3
OA C_8-diol ester	3	0	0	0	0	0	3	1	3	3	3	3	3	2	2	3	3	3	1	3	2	1	1	2	3	2	3
PTX2	3	0	0	0	0	0	3	3	3	3	3	3	3	3	3	3	3	3	3	3	3	3	3	3	3	3	3
PnTX E	3	0	0	0	0	0	3	3	3	3	3	3	3	3	3	3	3	3	3	3	3	3	3	3	3	3	3
PnTX F	3	0	0	0	0	0	3	3	3	3	3	3	3	3	3	3	3	3	3	3	3	3	3	3	3	3	3
PnTX G	3	0	0	0	0	0	3	3	3	3	3	3	3	3	3	3	3	3	3	3	3	3	3	3	3	3	3
**RP NEG**																											
16:0 OA ester	1	0	0	0	0	0	0	0	1	1	1	0	0	0	1	0	1	1	0	1	0	0	1	1	0	1	1
hYTX	2	0	0	0	0	0	1	0	0	1	1	0	1	0	0	0	0	1	0	1	0	0	0	0	0	0	3
DTX1	3	0	0	0	0	0	3	3	3	3	3	3	3	3	3	3	3	3	3	3	3	3	3	3	3	3	3
DTX2	3	0	0	0	0	0	3	3	3	3	3	3	3	3	3	3	3	3	3	3	3	3	3	3	3	3	3
OA	3	0	0	0	0	0	3	3	3	3	3	3	3	3	3	3	3	3	3	3	3	3	3	3	3	3	3
YTX	1	0	0	0	0	0	0	0	0	0	0	0	1	0	0	0	0	1	0	0	0	0	0	0	0	0	2
DA	2	0	0	0	0	0	0	0	0	0	0	0	0	0	0	0	0	0	0	1	0	0	0	0	1	1	2

LEGEND	
0	No precursor ion found
1	Precursor ion found
2	Precursor ion + 1 fragment ion found
3	Precursor ion + 2 fragment ions found
S	Standard solution
RP POS	Measurements with reversed phase LC and positive electrospray ionization
RP NEG	Measurements with reversed phase LC and negative electrospray ionization

Screening of lipophilic phycotoxins in food supplements

Spiking levels: Microcystins 30 µg L^{-1} for solids and 50 µg L^{-1} for liquids

Other lipophilic phycotoxins15 µg L^{-1}

Table S3.5. Main ingredient or ingredient of interest in samples used for validation food supplements.

Solid	Liquid
Microalgae powder	Oil from microalgae
Microalgae powder	Oil from microalgae
Chlorella powder	Oil from microalgae
Green lipped mussel	Fish oil
Chlorella	Fish oil
Green lipped mussel	Green lipped mussel
Green lipped mussel extract	Astaxanthine
Bladderwrack	Oyster extract
Oyster extract	Algae oil
Kelp	Red oil
Seaweed	Red krill oil
Chlorella	Green lipped mussel
Chlorella	Klamath blue green algae
Spirulina	Salmon oil
Chlorella	Omega 3 fish oil
Spirulina	Triple omega 3, 6, 9
Chlorella	Fish oil EPA50% DHA 25%
Vitamins	Fish oil
Chlorella	Multi vitamins
Green lipped mussel	Cod liver oil

Table S3.6. Validation results of lipophilic phycotoxins in solid food supplements.

RP POS	S	blanks					solid food supplements																				S
MC-HilR	3	0	0	0	0	2	2	2	0	0	0	0	0	0	0	2	0	0	0	0	0	0	0	0	0	3	3
MC-HtyR	3	0	0	0	0	3	0	2	3	0	3	2	0	3	0	3	0	0	0	0	0	0	0	0	0	3	3
MC-LA	3	0	0	0	0	0	0	0	0	0	1	0	0	2	0	0	0	0	0	0	0	0	0	0	2	0	3
MC-LF	3	0	0	2	0	0	0	0	0	0	0	0	0	1	0	1	0	0	0	0	0	0	0	0	0	2	3
MC-LR	3	0	0	0	0	0	1	1	0	0	0	0	0	1	2	1	0	0	0	0	0	0	0	0	0	2	3
MC-LW	3	0	0	0	0	0	0	0	0	0	0	0	0	0	0	3	2	0	0	0	0	0	0	0	0	3	3
MC-LY	3	0	0	0	0	0	0	0	0	0	0	0	0	3	0	2	0	0	0	0	0	0	0	0	0	3	3
MC-RR	3	0	0	0	0	0	2	2	3	2	1	1	1	2	2	3	2	1	1	2	1	2	1	3	1	3	3
MC-WR	3	0	0	0	0	0	0	1	0	0	1	0	0	0	0	2	2	0	0	0	0	0	1	0	2	3	3
MC-YR	3	0	0	0	0	0	1	0	3	0	0	0	0	3	0	3	0	0	0	0	0	0	0	0	3	3	3
NOD	3	0	0	0	0	0	1	1	3	2	1	2	1	2	1	3	3	1	1	0	1	1	1	0	1	3	3
13,19-didesMeSPXC	3	0	0	0	0	0	3	3	3	3	3	3	2	2	2	2	2	3	3	3	2	3	3	2	3	3	3
SPX1	3	0	0	0	2	0	2	2	2	2	2	3	3	2	2	2	3	3	2	2	2	2	2	2	3	3	3
20MeSPXG	3	0	0	0	0	0	2	3	3	2	2	3	3	3	3	2	3	3	2	2	2	1	2	3	3	3	3
Asp MC-LR	3	0	0	0	0	0	1	1	2	2	0	1	1	3	0	1	0	0	0	0	0	0	1	0	1	1	3
AZA1	3	0	0	0	0	0	2	1	2	1	0	2	3	1	3	1	2	1	0	1	0	1	1	1	1	3	3
AZA2	3	0	0	0	0	0	1	1	3	1	0	1	3	1	3	1	2	1	1	1	0	2	1	1	2	1	3
AZA3	3	0	1	0	0	0	1	1	2	1	0	3	3	2	3	1	2	0	0	1	0	1	0	1	1	2	3
AZA4	3	0	0	0	0	0	1	1	1	1	0	1	1	1	3	1	2	1	0	1	0	1	0	1	1	3	3
AZA5	3	0	0	0	0	0	2	1	1	1	1	1	2	1	2	1	1	1	0	1	0	1	0	1	1	1	3
GYM	3	0	3	0	2	0	2	2	3	1	3	3	3	3	1	2	1	3	2	1	1	2	2	1	3	3	3
OA methyl ester	3	0	0	0	0	0	1	1	1	2	0	1	1	1	2	1	2	0	0	0	1	1	0	0	0	2	2
OA C_8-diol ester	3	0	0	0	1	0	0	1	1	0	0	0	1	2	1	2	1	0	1	0	0	0	0	0	0	1	3
PTX2	3	0	0	0	0	0	1	1	2	1	2	0	2	2	2	2	2	2	0	1	0	2	1	0	2	1	3
PnTX E	3	0	0	0	0	0	2	2	2	2	2	2	2	2	0	2	2	2	2	2	2	2	2	2	2	3	3
PnTX F	3	0	0	0	0	0	2	2	3	2	2	2	2	2	2	2	3	2	2	2	1	1	2	2	2	3	3
PnTX G	3	0	0	0	0	0	3	2	3	3	2	3	3	3	3	3	3	3	2	2	1	2	2	3	3	3	3
**RP NEG**																											
16:0 OA ester	1	0	0	0	0	0	0	0	0	0	0	0	0	0	0	0	0	0	0	0	1	0	0	0	0	0	1
hYTX	3	0	0	0	0	0	0	0	0	0	0	0	0	0	0	0	0	0	0	0	0	0	0	0	0	0	3
DTX1	3	0	0	0	0	0	2	2	3	2	2	0	0	2	2	3	0	1	2	0	0	0	2	0	2	3	3
DTX2	3	0	0	0	0	0	2	3	2	2	0	0	2	3	2	3	3	0	0	0	0	0	0	1	1	2	3
OA	3	0	0	0	0	0	3	3	3	3	1	2	3	3	2	3	3	2	1	0	0	0	1	1	2	3	3
YTX	3	0	0	0	0	0	1	0	1	0	0	0	0	0	0	0	0	0	0	0	0	0	0	0	0	0	3

	LEGEND
0	No precursor ion found
1	Precursor ion found
2	Precursor ion + 1 fragment ion found
3	Precursor ion + 2 fragment ions found
S	Standard solution
RP POS	Measurements with reversed phase LC and positive electrospray ionization
RP NEG	Measurements with reversed phase LC and negative electrospray ionization

Table S3.7. Validation results of lipophilic phycotoxins in liquid food supplements.

RP POS	S	blanks					liquid food supplements																				S
MC-HilR	3	0	0	0	0	0	3	3	3	3	3	3	3	3	3	0	0	3	3	3	3	3	3	3	0	3	3
MC-HtyR	3	0	0	0	0	0	3	3	3	3	3	3	3	3	3	0	3	3	3	3	3	3	3	3	0	3	3
MC-LA	3	0	0	0	0	0	3	3	3	3	3	0	3	2	3	0	0	0	2	3	0	3	3	3	0	3	3
MC-LF	3	0	0	0	0	0	3	3	3	3	3	0	3	3	3	0	0	0	3	3	3	3	3	3	2	3	3
MC-LR	3	0	0	0	0	0	3	3	3	3	3	3	3	3	3	0	0	1	2	3	3	3	3	3	2	3	3
MC-LW	3	0	0	0	0	0	3	3	3	3	3	0	3	3	3	0	0	0	3	3	3	3	0	3	2	3	3
MC-LY	3	0	0	0	0	0	3	3	3	3	3	0	3	2	3	0	0	0	3	3	0	3	0	3	2	3	3
MC-RR	3	0	0	0	0	0	3	3	3	3	3	3	3	3	3	3	3	3	3	3	3	3	3	3	0	3	3
MC-WR	3	0	0	0	0	0	3	3	3	3	3	3	3	2	3	0	3	3	3	3	3	3	3	3	0	3	3
MC-YR	3	0	0	0	0	0	3	3	3	3	3	3	3	3	3	0	0	3	3	3	3	3	3	3	0	3	3
NOD	3	0	0	0	0	0	3	3	3	3	3	3	3	3	3	0	2	3	2	3	3	3	3	3	2	3	3
13,19-didesMeSPXC	3	0	0	0	0	0	3	3	3	3	3	3	3	3	3	3	3	3	3	3	3	3	3	3	3	3	3
SPX1	3	0	0	0	0	0	3	3	3	3	3	3	3	3	3	3	3	3	3	3	3	3	3	3	3	3	3
20MeSPXG	3	0	0	0	0	0	3	3	3	3	3	3	3	3	3	2	3	3	3	3	3	3	3	3	3	3	3
Asp MC-LR	3	0	0	0	0	0	3	3	3	3	3	3	3	3	3	0	3	1	1	3	3	3	3	3	1	3	3
AZA1	3	0	0	0	0	0	3	3	3	3	3	1	3	2	3	0	1	1	3	3	3	1	3	3	2	3	3
AZA2	3	0	0	0	0	0	3	3	3	3	3	2	3	2	3	1	1	1	3	3	1	3	3	3	1	3	3
AZA3	3	0	0	0	0	0	3	3	3	3	3	2	3	3	3	1	1	1	3	3	2	2	3	3	3	3	3
AZA4	3	0	0	0	0	0	3	3	3	3	3	3	1	3	3	0	0	1	3	3	3	3	3	3	1	3	3
AZA5	3	0	0	0	0	0	3	3	3	3	3	3	1	1	3	1	1	1	3	3	3	3	3	3	1	3	3
GYM	3	0	0	0	0	0	3	3	3	3	3	3	3	3	3	3	3	3	1	3	3	3	3	3	3	3	3
OA methyl ester	2	0	0	0	0	0	2	2	3	2	2	1	1	1	2	0	0	1	2	2	0	0	0	2	1	2	2
OA C_8-diol ester	3	0	0	0	0	0	2	1	2	2	3	1	3	3	2	0	1	1	2	2	0	0	0	2	1	2	3
PTX2	3	0	0	0	0	0	3	3	3	3	3	2	1	2	3	0	0	1	3	3	2	2	2	3	1	3	3
PnTX E	3	0	0	0	0	0	3	3	3	3	3	3	3	3	3	0	2	2	2	3	3	3	3	3	2	3	3
PnTX F	3	0	0	0	0	0	3	3	3	3	3	3	3	3	3	2	2	2	3	3	3	3	3	3	2	3	3
PnTX G	3	0	0	0	0	0	3	3	3	3	3	3	3	3	3	2	3	3	3	3	3	3	3	3	3	3	3
RP NEG																											
16:0 OA ester	1	0	0	0	0	0	0	0	0	0	0	0	1	0	0	0	0	0	0	0	0	0	0	0	0	0	0
hYTX	3	0	0	0	0	0	3	3	3	2	2	0	0	0	3	0	0	0	1	2	1	1	1	2	0	1	2
DTX1	3	0	0	0	0	0	3	3	3	3	3	0	3	3	0	0	0	0	3	3	3	3	3	3	0	3	3
DTX2	3	0	0	0	0	0	3	3	3	3	3	2	3	3	3	0	2	3	3	3	3	3	3	3	1	3	3
OA	3	0	0	0	0	0	3	3	3	3	3	3	3	3	3	0	0	3	3	3	3	3	3	3	0	3	3
YTX	3	0	0	0	0	0	2	2	2	1	1	0	0	0	2	0	0	0	1	2	1	0	1	1	0	0	2

LEGEND	
0	No precursor ion found
1	Precursor ion found
2	Precursor ion + 1 fragment ion found
3	Precursor ion + 2 fragment ions found
S	Standard solution
RP POS	Measurements with reversed phase LC and positive electrospray ionization
RP NEG	Measurements with reversed phase LC and negative electrospray ionization

Table S3.8. summary Validated levels screening.

Compound	(Shell) fish (µg kg-1)	Water (µg L-1)	Food supplements solids (µg kg-1)	Food supplements liquids (µg kg-1)
**HILIC POS**				
ATX	600	120	n.p.	n.p.
dcNEO	600	120	n.p.	n.p.
dcSTX	600	120	n.p.	n.p.
NEO	600	120	n.p.	n.p.
STX	600	120	n.p.	n.p.
TTX	600 (except ensis)	120	n.p.	n.p.
**HILIC NEG**				
CYN	*	120	n.p.	n.p.
dcGTX2	600	120	n.p.	n.p.
dcGTX3	134	27	n.p.	n.p.
DA	600	*	n.p.	n.p.
GTX1&4	795	159	n.p.	n.p.
GTX2	600	120	n.p.	n.p.
GTX3	228	46	n.p.	n.p.
GTX5	600	120	n.p.	n.p.
C1	600 (except mussel)	120	n.p.	n.p.
C2	*	*	n.p.	n.p.
**RP POS**				
MC-HilR	150	10	*	*
MC-HtyR	150	10	*	*
MC-LA	150	10	*	*
MC-LF	150	10	*	*
MC-LR	150	10	*	*
MC-LW	150	10	*	*
MC-LY	150	10	*	*
MC-RR	150	10	*	50
MC-WR	150	10	*	*
MC-YR	150	10	*	*
NOD	150	10	*	50
13,19-didesMeSPXC	80	5	15	15
SPX1	80	5	15	15
20MeSPXG	80	5	15	15
Asp MC-LR	150	10	*	*
AZA1	80	5	*	*
AZA2	80	5	*	*
AZA3	80	5	*	*
AZA4	80	5	*	*
AZA5	80 (except fish)	5	*	*
GYM	80	5	*	15
OA methyl ester	80	5	*	*
OA C_8-diol ester	80	*	*	*
PTX2	80	5	*	*

PnTX E	80	5	15	15
PnTX F	80	5	15	15
PnTX G	80	5	15	15
**RP NEG**				
16:0 OA ester	*	*	*	*
hYTX	80	*	*	*
DTX1	80	5	*	*
DTX2	80	5	*	*
OA	80	5	*	*
YTX	*	*	*	*

* no satisfactory results

n.p. not performed

Quantitation of ASP, PSP and DSP in tissue

Table S3.9. spike levels of ASP, PSP and DSP for quantitative validation in shellfish.

Compound	Toxin group	0.5 (ug kg^{-1})	1 (ug kg^{-1})	Matrix matched standards (ug kg^{-1})
DA	ASP	10,000	20,000	0, 5,000, 10,000, 20,000, 50,000
STX, dcSTX, NEO, dcNEO, GTX1&4[1], GTX2&3[1], GTX5, dcGTX2&3[1]	PSP	400	800	0, 400, 600, 800, 1200
OA, DTX1, DTX2	DSP	80	160	0, 20, 40, 80, 160, 240
AZA1, AZA2, AZA3		80	160	0, 20, 40, 80, 160, 240
YTX, hYTX		250	500	0, 62.5, 125, 250, 500, 750
SPX1		200	400	0, 50, 100, 200, 400, 600
GYM		100	200	0, 25, 50, 100, 200, 300
PnTX G		25	50	0, 6.25, 12.5, 25, 50, 75

[1] Concentrations of the highest isomer present given.

Table S3.10. Validation results of lipophilic phycotoxins in tissue, MMS before samples, recovery and repeatability.

Compound	Linearity	% Deviation from the back-calculated concentration Level 1	Level 2	Level 3	Level 4	Level 5	Recovery	RSD_r 0.5	RSD_r 1
**Requirements**	**>0.9900**	**<20%**	**<20%**	**<20%**	**<20%**	**<20%**	**70-120%**	**<20%**	**<20%**
OA	0.999	3.3%	-4.0%	-7.0%	3.1%	-0.5%	120.0%	17.1%	*7.7%
DTX1	0.999	-9.6%	8.3%	-4.1%	-3.8%	2.0%	109.8%	4.6%	8.5%
DTX2	0.999	-20.8%	-3.9%	6.2%	1.3%	-1.0%	111.4%	15.0%	12.0%
YTX	0.995	-20.6%	-10.5%	-14.6%	4.5%	0.1%	90.1%	*10.7%	18.1%
hYTX	0.994	6.6%	-9.1%	-21.0%	-1.2%	3.1%	75.2%	*7.1%	5.5%
AZA1	0.998	10.3%	7.9%	-2.3%	-7.2%	3.1%	95.0%	5.1%	3.0%
AZA2	0.999	12.1%	-1.6%	-2.7%	3.5%	-1.3%	88.7%	5.5%	3.4%
AZA3	1.000	3.7%	5.8%	-1.4%	-0.3%	0.1%	108.1%	6.9%	4.8%
SPX1	0.999	-1.1%	1.1%	-0.7%	4.5%	-1.9%	109.9%	5.0%	7.9%
GYM	1.000	2.6%	2.5%	-0.8%	-1.5%	0.7%	102.3%	5.6%	6.5%
PnTX G	0.999	-4.2%	-1.3%	-1.8%	3.9%	-1.5%	102.2%	7.1%	9.8%

* One outlier, tested with Grubbs test, removed from dataset

Table S3.11. Validation results of lipophilic phycotoxins in tissue, MMS after samples.

Compound	Linearity	% Deviation from the back-calculated concentration					Drift in sensitivity
		Level 1	Level 2	Level 3	Level 4	Level 5	
**Requirements**	**>0.9900**	**<20%**	**<20%**	**<20%**	**<20%**	**<20%**	**<30%**
OA	0.994	-1.4%	-13.0%	0.1%	11.6%	-4.8%	-24.2%
DTX1	0.997	-13.3%	3.3%	-10.1%	8.1%	-2.5%	-15.7%
DTX2	0.999	-12.8%	-5.3%	4.6%	2.6%	-1.4%	6.4%
YTX	0.993	-0.8%	-5.8%	-22.4%	-1.0%	3.1%	11.4%
hYTX	0.995	3.4%	-6.5%	-15.1%	-4.9%	4.0%	3.4%
AZA1	0.995	-3.0%	-10.2%	4.5%	10.0%	-4.6%	-15.5%
AZA2	0.998	3.0%	-4.0%	-1.9%	6.9%	-2.8%	-4.3%
AZA3	0.997	-9.1%	4.8%	3.9%	6.6%	-3.4%	-4.3%
SPX1	1.000	-0.2%	0.0%	-3.0%	-0.4%	0.5%	8.8%
GYM	0.998	15.0%	3.9%	-6.7%	-5.0%	2.8%	9.1%
PnTX G	0.999	-6.9%	-1.7%	5.9%	-4.9%	1.6%	1.4%

Table S3.12. Validation results of hydrophilic phycotoxins in tissue, MMS before samples, recovery and repeatability.

Compound	Linearity	% Deviation from the back-calculated concentration				Recovery	RSD_r 0.5	RSD_r 1
		Level 1	Level 2	Level 3	Level 4			
Requirements	>0.9900	<20%	<20%	<20%	<20%	70-120%	<20%	<20%
DA	0.994	-25.0%	9.6%	13.3%	-3.4%	93.0%	3.4%	1.4%
STX	0.994	-16.9%	1.9%	8.5%	-2.4%	39.2%	59.9%	42.7%
dcSTX	0.999	0.6%	3.2%	2.1%	-1.8%	33.2%	53.8%	37.4%
NEO	0.966	-8.9%	-31.3%	3.7%	7.2%	18.4%	69.1%	43.8%
dcNEO	0.992	-12.4%	-12.0%	5.2%	2.1%	9.2%	50.0%	35.2%
GTX1&4	0.981	-23.7%	-15.0%	3.2%	5.0%	85.2%	8.4%	12.5%
GTX2	0.995	-7.2%	-3.9%	-5.3%	4.1%	95.9%	7.9%	3.8%
GTX3	0.999	-3.6%	5.3%	-0.7%	-0.6%	106.2%	8.8%	7.2%
GTX5	0.994	-14.3%	-6.4%	-0.9%	3.6%	92.5%	5.7%	4.6%
dcGTX2	0.994	-17.1%	-0.4%	-2.1%	2.9%	57.3%	22.1%	12.9%
dcGTX3	0.994	-16.9%	-5.6%	2.0%	2.4%	83.8%	13.8%	8.5%
C1	0.996	-9.5%	-7.3%	4.1%	1.0%	81.9%	21.5%	8.3%
C2	0.979	-11.2%	-21.9%	0.8%	6.4%	38.3%	*6.0%	30.0%

* One outlier, tested with Grubbs test, removed from dataset

Table S3.13. Validation results of hydrophilic phycotoxins in tissue, MMS after samples.

Compound	Linearity	% Deviation from the back-calculated concentration				Drift in sensitivity
		Level 1	Level 2	Level 3	Level 4	
**Requirements**	**>0.9900**	**<20%**	**<20%**	**<20%**	**<20%**	**<30%**
DA	0.993	-27.1%	8.9%	16.3%	-3.8%	-15.2%
STX	0.988	-11.2%	-16.0%	1.3%	4.6%	-17.5%
dcSTX	0.997	-9.3%	-5.0%	0.6%	2.0%	-18.1%
NEO	0.968	-32.3%	-16.6%	0.1%	7.7%	-25.3%
dcNEO	0.969	-27.1%	-16.1%	-4.8%	9.2%	-14.1%
GTX1&4	0.980	-20.1%	-13.9%	-3.2%	7.1%	-10.4%
GTX2	0.994	-7.6%	-10.6%	-0.8%	3.8%	-11.5%
GTX3	0.996	-6.6%	-8.7%	-0.6%	3.2%	-5.9%
GTX5	0.995	-10.3%	-9.3%	1.6%	2.7%	-8.5%
dcGTX2	0.982	-26.5%	-2.9%	-6.7%	6.6%	-4.6%
dcGTX3	0.994	-17.0%	-4.3%	0.5%	2.8%	-6.8%
C1	0.996	-5.8%	7.1%	-7.3%	2.1%	-15.8%
C2	0.991	-23.0%	-2.0%	-1.0%	3.5%	-16.2%

S4: Standards

Table S4.1. Hydrophilic standards.

Chemicals	Abbreviation	Concentration or purity	Supplier
L-2-Amino-3-methylaminopropionic acid	BMAA	>97%	Sigma-Aldrich[1]
Anatoxin	ATX	4.96 ± 0.18 μg mL^{-1}	NRC[2]
Cylindrospermopsin	CYN	12.6 ± 0.8 μg mL^{-1}	NRC[2]
Decarbamoylgonyautoxin 2&3	dcGTX2	40.9 ± 1.8 μg mL^{-1}	NRC[2]
	dcGTX3	9.2 ± 0.3 μg mL^{-1}	
Decarbamoylsaxitoxin	dcSTX	16.7 ± 0.5 μg mL^{-1}	NRC[2]
Decarbamoylneosaxitoxin	dcNEO	8.0 ± 0.3 μg mL^{-1}	NRC[2]
L-2,4-Diaminobutyric acid	DAB	>95%	Sigma-Aldrich[1]
Domoic acid	DA	>90%	Sigma-Aldrich[1]
Gonyautoxin 1&4	GTX1	24.8 ± 1.3 μg mL^{-1}	NRC[2]
	GTX4	8.1 ± 0.7 μg mL^{-1}	
Gonyautoxin 2&3	GTX2	45.2 ± 2.3 μg mL^{-1}	NRC[2]
	GTX3	17.2 ± 0.9 μg mL^{-1}	
Gonyautoxin 5	GTX5	24.7 ± 1.1 μg mL^{-1}	NRC[2]
Neosaxitoxin	NEO	20.7 ± 1.1 μg mL^{-1}	NRC[2]
Saxitoxin	STX	19.8 ± 0.4 μg mL^{-1}	NRC[2]
N-Sulfocarbamoylgonyautoxin-2&3	C1	53.9 ± 1.8 μg mL^{-1}	NRC[2]
	C2	16.1 ± 1.3 μg mL^{-1}	
Tetrodotoxin	TTX	96%	Latoxan[3]
Tetrodotoxin & 4,9-anhydro TTX	TTX	25.6 ± 1.8 μg mL^{-1}	CIFGA[4]
	anhTTX	3.0 ± 0.2 μg mL^{-1}	

[1] Sigma-Aldrich, Zwijndrecht, The Netherlands

[2] National Research Council, Measurement science and standards, Halifax, Canada

[3] Latoxan, Valence, France

[4] CIFGA, Lugo, Spain

Table S4.2. lipophilic standards.

Chemicals	Abbreviation	Concentration or purity	Supplier
Azaspiracid-1	AZA1	1.24 ± 0.07 µg mL^{-1}	NRC[1]
Azaspiracid-2	AZA2	1.28 ± 0.05 µg mL^{-1}	NRC[1]
Azaspiracid-3	AZA3	1.04 ± 0.04 µg mL^{-1}	NRC[1]
Azaspiracid-4	AZA4	1.19 ± 0.07 µg mL^{-1}	CIFGA[2]
Azaspiracid-5	AZA5	1.20 ± 0.07 µg mL^{-1}	CIFGA[2]
Brevetoxin 2	PbTx 2	95%	Latoxan[3]
Brevetoxin 3	PbTx 3	95%	Latoxan[3]
Brevetoxin 9	PbTx 9	95%	Latoxan[3]
13-Desmethyl spirolide C	SPX1	7.0 ± 0.4 µg mL^{-1}	NRC[1]
13,19-Didesmethyl spirolide C	13,19-didesMeSPXC	10.24 ± 0.98 µg mL^{-1}	CIFGA[2]
Dinophysistoxin-1	DTX1	15.1 ± 1.1 µg mL^{-1}	NRC[1]
Dinophysistoxin-2	DTX2	7.8 ± 0.4 µg mL^{-1}	NRC[1]
Gymnodimine	GYM	5.0 ± 0.2 µg mL^{-1}	NRC[1]
Homoyessotoxin	hYTX	5.8 ± 0.3 µg mL^{-1}	NRC[1]
20-Methyl spirolide G	20MeSPXG	7.01 ± 0.61 µg mL^{-1}	CIFGA[2]
Microcystin-HilR	MC-HilR	>95%	Enzo Life Sciences[4]
Microcystin-HtyR	MC-HtyR	>95%	Enzo Life Sciences[4]
Microcystin-LA	MC-LA	>95%	Enzo Life Sciences[4]
Microcystin-LF	MC-LF	>95%	Enzo Life Sciences[4]
Microcystin-LR	MC-LR	>95%	Enzo Life Sciences[4]
[D-Asp^3]Microcystin-LR	Asp MC-LR	>95%	Enzo Life Sciences[4]
Microcystin-LW	MC-LW	>95%	Enzo Life Sciences[4]
Microcystin-LY	MC-LY	>95%	Enzo Life Sciences[4]
Microcystin-RR	MC-RR	>95%	Enzo Life Sciences[4]
Microcustin-YR	MC-YR	>95%	Enzo Life Sciences[4]
Nodularin	NOD	>95%	Enzo Life Sciences[4]
Okadaic acid	OA	13.7 ± 0.6 µg mL^{-1}	NRC[1]
Okadaic acid C_8-diol ester	OA C_8-diol ester	>90%	Enzo Life Sciences[4]
Okadaic acid methyl ester	OA methyl ester	>90%	Enzo Life Sciences[4]
Pacific ciguatoxin 1	pCTX1	No certified concentration	University of Queensland[5]
Pacific ciguatoxin 2	pCTX2	No certified concentration	University of Queensland[5]
Pacific ciguataxin 3	pCTX3	No certified concentration	University of Queensland[5]
7-O-Palmitoylokadaic acid	16:0 OA ester, DTX3	90-94%	MP Biomedicals[6]
Palytoxin	PlTX	>90%	Wako[7]
Pectenotoxin-2	PTX2	8.6 ± 0.3 µg mL^{-1}	NRC[1]
Pinnatoxin E	PnTX E	No certified concentration	Cawthron Institute[8]
Pinnatoxin F	PnTX F	No certified concentration	Cawthron Institute[8]
Pinnatoxin G	PnTX G	No certified concentration	Cawthron Institute[8]
Yessotoxin	YTX	5.6 ± 0.2 µg mL^{-1}	NRC[1]

[1] National Research Council, Measurement science and standards, Halifax, Canada

[2] CIFGA, Lugo, Spain

[3] Latoxan, Valence, France

[4] Enzo Life Sciences, Antwerp, Belgium

[5] Professor Lewis, Institute for molecular Bioscience, The University of Queensland, Australia

[6] MP Biomedicals, Santa Ana, United states

[7] Wako, Osaka, Japan

[8] Cawthron Institute, Nelson, New Zealand
